# Changes in physiological activities and root exudation profile of two grapevine rootstocks reveal common and specific strategies for Fe acquisition

**DOI:** 10.1038/s41598-020-75317-w

**Published:** 2020-11-02

**Authors:** Laura Marastoni, Luigi Lucini, Begoña Miras-Moreno, Marco Trevisan, Davide Sega, Anita Zamboni, Zeno Varanini

**Affiliations:** 1 Biotechnology Department, University of Verona, Verona, Italy; 2 Department for Sustainable Food Process, University Cattolica del Sacro Cuore, Piacenza, Italy

**Keywords:** Physiology, Plant sciences

## Abstract

In several cultivation areas, grapevine can suffer from Fe chlorosis due to the calcareous and alkaline nature of soils. This plant species has been described to cope with Fe deficiency by activating *Strategy I* mechanisms, hence increasing root H^+^ extrusion and ferric-chelate reductase activity. The degree of tolerance exhibited by the rootstocks has been reported to depend on both reactions, but to date, little emphasis has been given to the role played by root exudate extrusion. We studied the behaviour of two hydroponically-grown, tolerant grapevine rootstocks (Ramsey and 140R) in response to Fe deficiency. Under these experimental conditions, the two varieties displayed differences in their ability to modulate morpho-physiological parameters, root acidification and ferric chelate reductase activity. The metabolic profiling of root exudates revealed common strategies for Fe acquisition, including ones targeted at reducing microbial competition for this micronutrient by limiting the exudation of amino acids and sugars and increasing instead that of Fe(III)-reducing compounds. Other modifications in exudate composition hint that the two rootstocks cope with Fe shortage via specific adjustments of their exudation patterns. Furthermore, the presence of 3-hydroxymugenic acid in these compounds suggests that the responses of grapevine to Fe availability are rather diverse and much more complex than those usually described for *Strategy I* plants.

## Introduction

Despite its great abundance in the Earth’s crust, the bioavailability of iron (Fe) is sharply reduced under aerobic conditions, especially in high-pH and calcareous soils^1^. Fe is the micronutrient required in the largest amount by plants^2^ and plays a role in a variety of metabolic processes such as respiration, photosynthesis and chlorophyll biosynthesis^1,2^. As a consequence, its shortage can have a negative impact on plant growth and crop productivity, given that one-third of the world's land surface area is estimated to be calcareous^3^. It is generally accepted that plants cope with low Fe bioavailability using two different mechanisms of acquisition, commonly referred to as *Strategy I* and *Strategy II*^2^. When Fe is lacking, dicots and non-graminaceous monocots activate the former mechanism, consisting of enhanced H^+^ extrusion, reduction of Fe(III) to Fe(II) and uptake of Fe(II)^4^ brought about by the increased activity of root plasma membrane (PM) H^+^-ATPases, ferric-chelate reductase oxidases (FRO) and iron-regulated transporters (IRT), respectively^2,4^. In the chelation-based *Strategy II*, confined to graminaceous species, there is an increased release of phytosiderophores (PSs) and mugineic acids (MAs) into the rhizosphere mediated by translocase of the outer membrane (TOM)^2^ complexes. MAs have a high affinity for Fe(III) and the roots take up the Fe-PS complexes from the rhizosphere via the YELLOW STRIPE transporter (YS)^2,4^. Despite the clear distinction usually made between graminaceous and non-graminaceous species for the type of strategy employed, the picture is not so clear-cut. Roots of *Strategy I* plants have been described to exude metabolites such as phenolics that may play a role in Fe uptake by acting as reducing and/or chelating agents^5^, whilst Fe-starved *Strategy II* species such as rice and maize have been observed to induce IRT-encoding genes^6–8^. For this reason, a combined strategy has been recently postulated for the uptake of this micronutrient by grass species^8,9^.

Cultivated grapevines are usually a combination of two separate genotypes, i.e. a scion (cultivar) grafted onto a rootstock. The cultivars, mainly belonging to the species *Vitis vinifera,* are selected for their fruiting performance. However, it is the rootstocks (individual *Vitis* species or crosses between two or more species) that provide the water and nutrients, taking them up from the soil^10,11^. Rootstocks are classified as susceptible or tolerant to lime-induced Fe deficiency^11^ and several works confirm that grapevines possess the same *Strategy I* mechanisms that are typical of other dicots, i.e. increased H^+^ release and ferric chelate reductase activity (FCR)^10^. These two root activities were proposed as a useful method to screen genotypes for their response to bicarbonate-induced chlorosis^12^ and Fe shortage in hydroponic cultures^13^. Assimakopoulou et al.^14^ on the other hand suggest that the observation of chlorotic symptoms and plant growth parameters might be a more useful screening tool than measuring leaf Fe content and root ferric chelate reductase (FCR) activity. The release of root exudates into the rhizosphere can directly or indirectly improve Fe acquisition and may therefore be of crucial importance when attempting to discriminate genotypes according to their ability to cope with low Fe availability. After the pioneering works carried out in the ‘80s^5,15^, more recent metabolomic investigations support the idea a role that secondary metabolites, and coumarins in particular^16–20^, can play a major role in the plants’ response to Fe deficiency. However, there is no literature available on the characterization of root exudates by grapevine, with the sole exception of a paper describing the changes in organic (oxalic, tartaric, malic and ascorbic) acids exuded by Fe-starved 140R and after treatment with Fe-EDDHA and Fe-heme^21^.

Aim of our work was to shed more light on the responses of two grapevine rootstocks, Ramsey (*Vitis champini*) and 140 Ruggeri (140R*, Vitis berlandieri* × *Vitis rupestris*), to Fe shortage. Both rootstocks are classified as tolerant to lime-induced chlorosis^11,22,23^, although some authors describe Ramsey as susceptible^24^. We investigated the mechanisms used by these two rootstocks to respond to Fe shortage, comparing changes in morphological, physiological and biochemical parameters (H^+^ extrusion and FCR). A comprehensive metabolomic profiling of the root exudates was also carried out, to better understand the rootstock-mediated exudation patterns as a function of Fe availability. Taken together, these data suggest that, even though they belong to the same genus, grapevines can display quite a diverse array of mechanisms for Fe uptake from the rhizosphere.

## Results

### Responses to Fe deficiency

**Figure 1:**
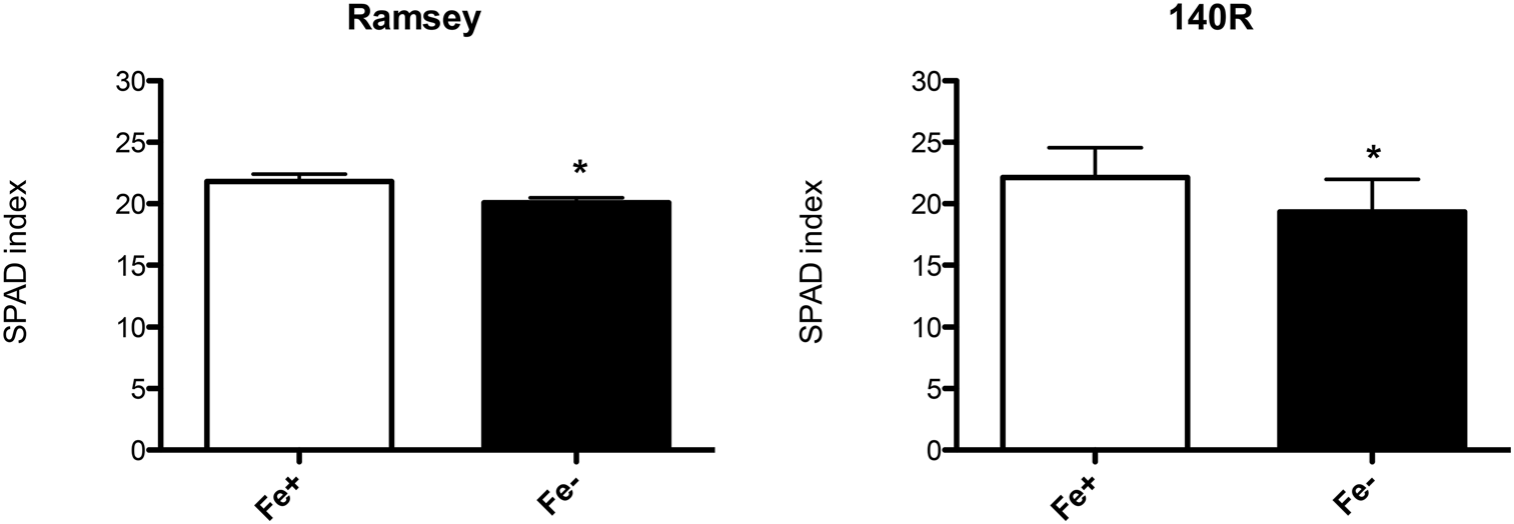
SPAD values in Fe-sufficient and Fe-deficient microcuttings of Ramsey and 140R. The statistical significance was determined by paired Student’s t-test (mean ± SE, n = 3 independent growth experiments, *P < 0.05).

**Figure 2:**
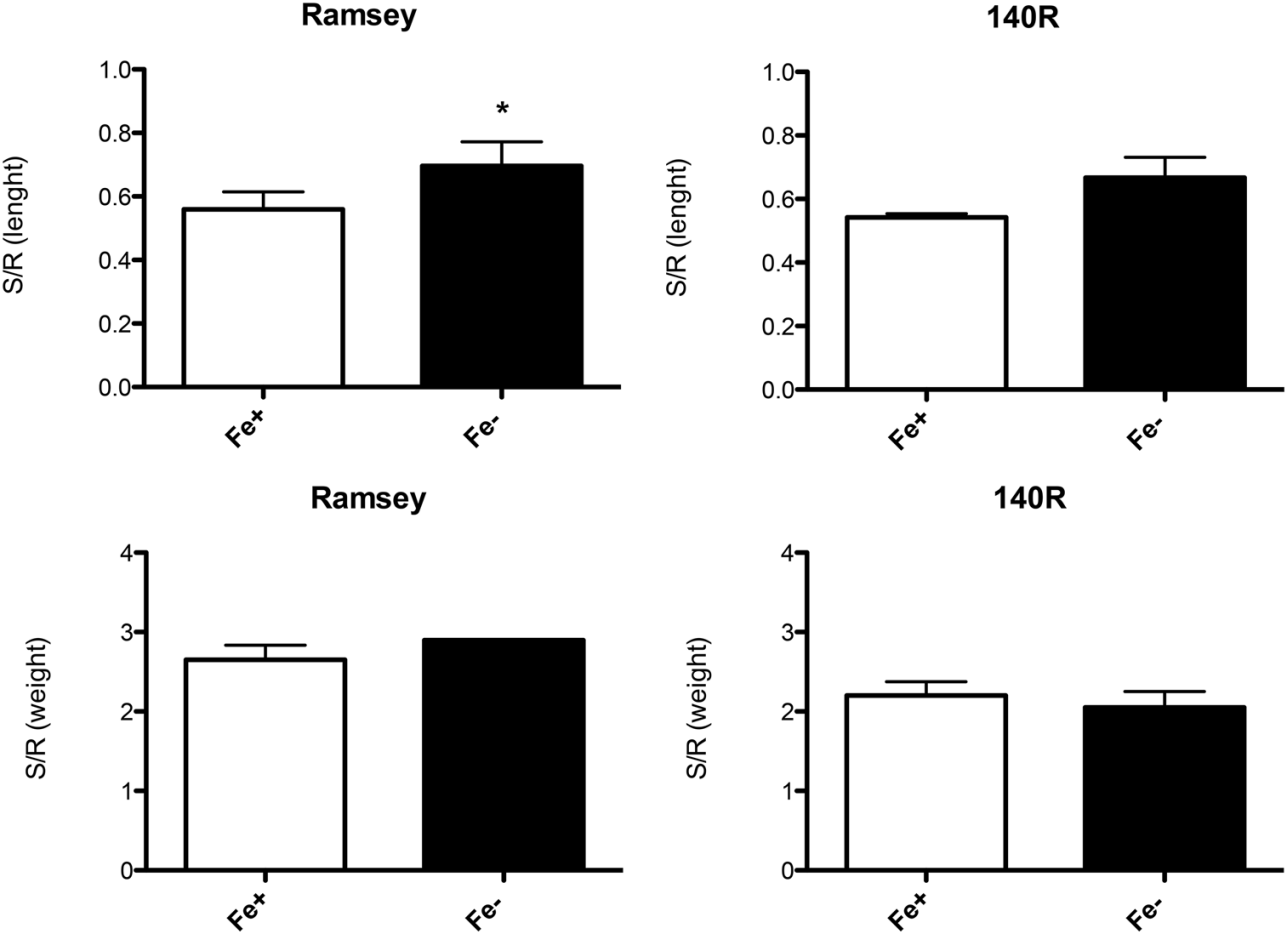
Shoot/root length ratio and shoot/root fresh weight ratio in Fe-sufficient and Fe-deficient microcuttings of Ramsey and 140R. The statistical significance was determined by paired Student’s t-test (mean ± SE, n = 3 independent growth experiments, *P < 0.05).

**Figure 3:**
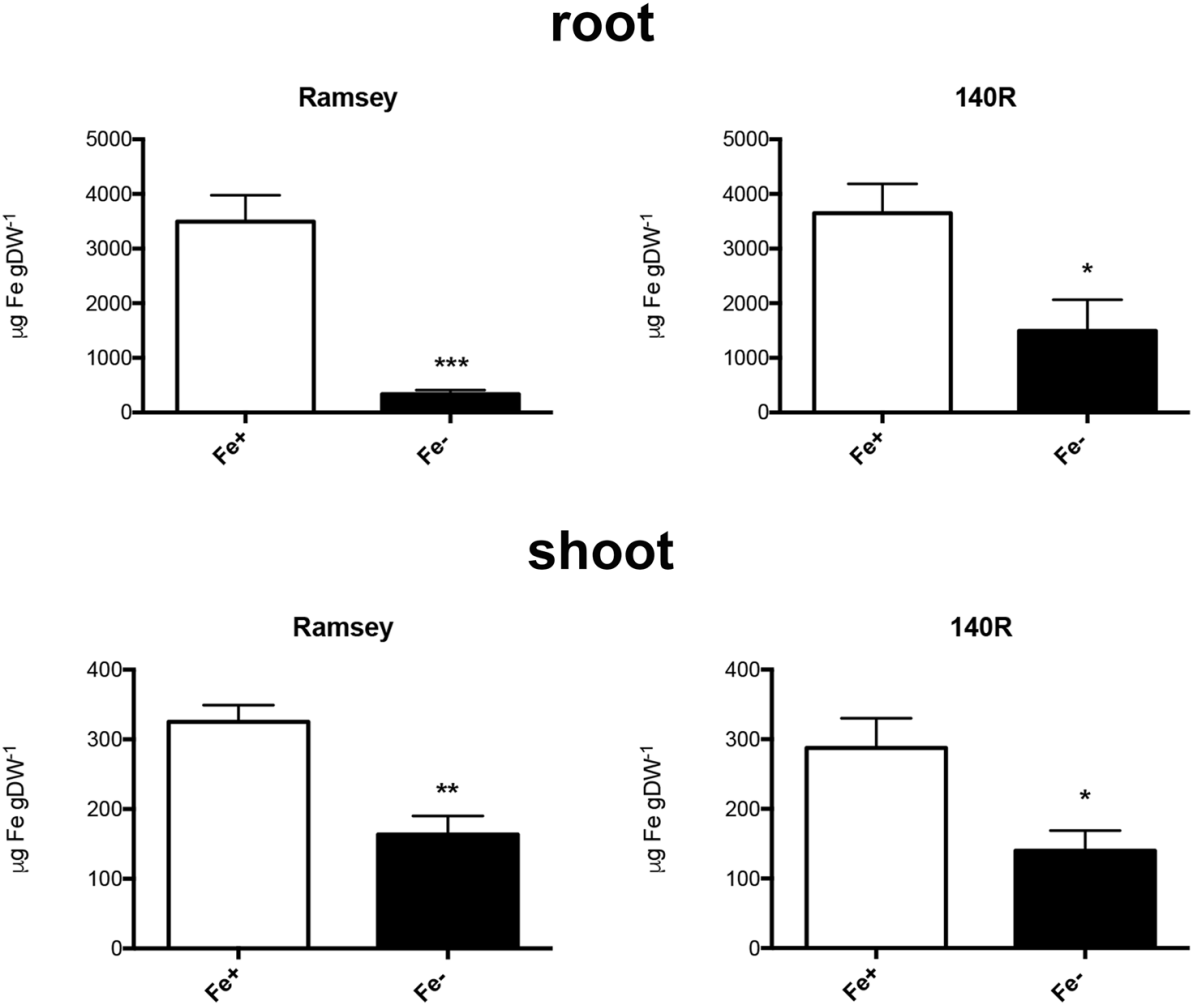
Fe content determined by ICP-MS in root and shoot tissues of Fe-sufficient (Fe+) and Fe-deficient (Fe−) microcuttings of Ramsey and 140R. The statistical significance was determined by Student’s t-test (mean ± SE, n = 6 plants, *P < 0.05, ***P < 0.001).

The symptoms of Fe deficiency were evaluated on Ramsey and 140R microcuttings by measuring morphological and biochemical parameters when Fe chlorosis symptoms were clearly visible on the apical leaves of both genotypes (35 days of growth in the absence of Fe, Supplementary Fig. S1). In particular, SPAD (Soil–Plant Analysis Development) index values, root and shoot length and fresh weight, Fe content in tissues, in vivo H^+^ extrusion and ferric-chelate reduction were determined both in Fe-sufficient (NS with Fe, 30 μM, Fe +) and Fe-deficient (NS without Fe, 0 μM, Fe−) microcuttings of both genotypes. As expected, Ramsey and 140R plants grown in the absence of the micronutrient both displayed significantly lower SPAD index values than the respective Fe-sufficient control plants (Fig. 1). The two genotypes however displayed some differences when the growth parameters were taken into account. As regards the shoot to root length ratio, Fe shortage only caused a statistically significant increase of this parameter in Ramsey (Fig. 2, Supplementary Fig. S1), whereas under the same conditions, neither genotype displayed significant changes in their shoot to root fresh weight ratio (Fig. 2). Fe shortage also reduced Fe content in the root and shoot tissues of both rootstocks, with the greatest difference measured in the roots of Ramsey microcuttings (Fig. 3).

**Figure 4:**
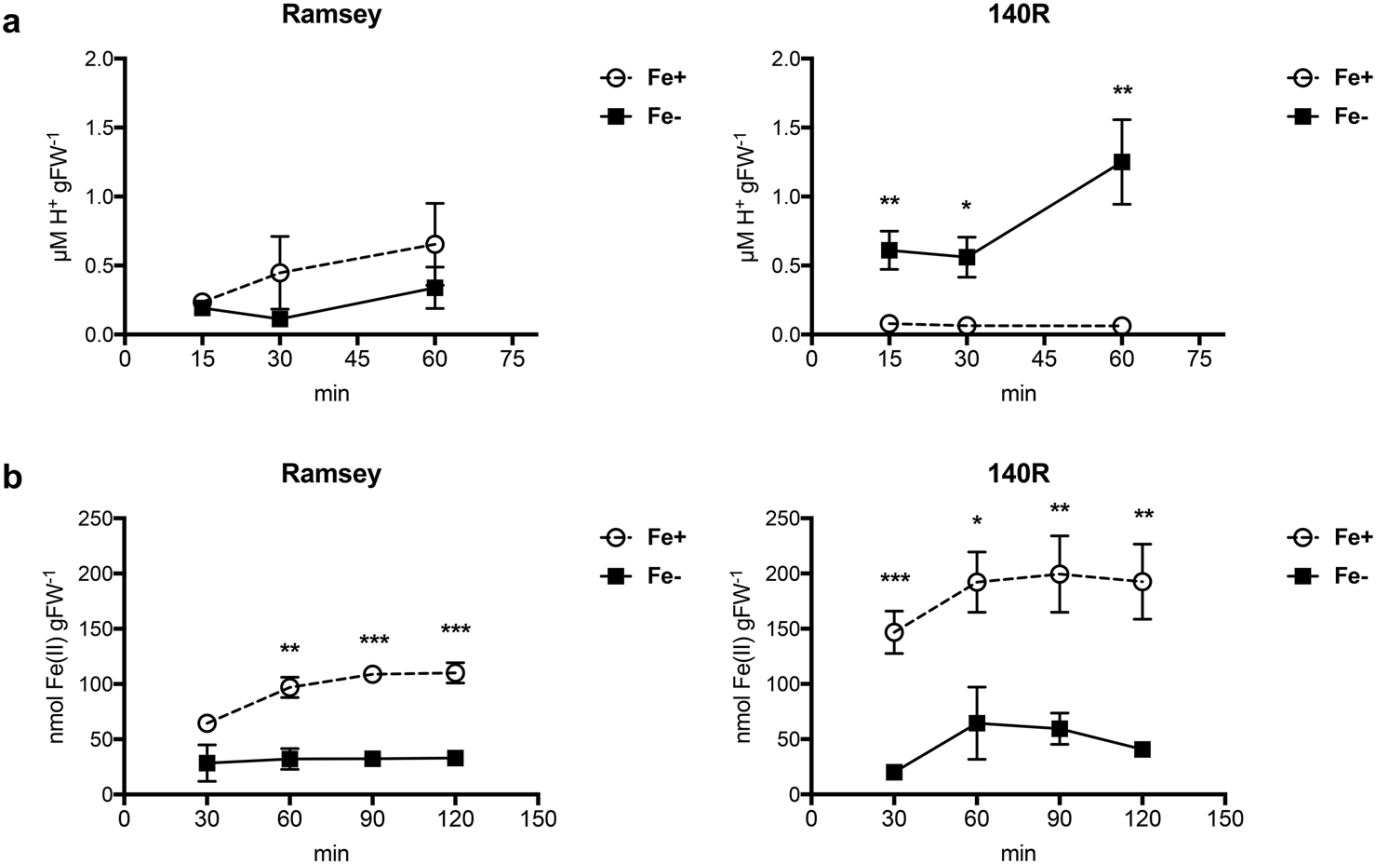
(**a**) H^+^ extrusion from roots of Fe-sufficient (Fe+) and for Fe-deficient (Fe−) microcuttings of Ramsey and 140R measured at 15, 30 and 60 min. (**b**) Time-course of root FCR activity. Reduction of Fe(III)-EDTA by the root apparatus measured after 30, 60, 90 and 120 min and expressed as nmol Fe(II) gFW-1 in Fe-sufficient (Fe+) and for Fe-deficient (Fe−) microcuttings of Ramsey and 140R. The statistical significance was determined by Student’s t-test (mean ± SE, n = 4 plants, *P < 0.05, **P < 0.01, ***P < 0.001).

As far as the typical *Strategy I* root responses were concerned, we observed that under Fe deficiency conditions, 140R rootstocks significantly enhanced H^+^ extrusion activity whilst no such differences emerged in Ramsey microcuttings (Fig. 4a). As regards the root ferric-chelate reduction (FCR) activity, significantly greater levels were measured in the Fe-sufficient plants, irrespective of the genotype (Fig. 4b). The highest values however were observed in 140R microcuttings, irrespective of their nutritional status (Fig. 4b and Supplementary Fig. S2).

### Metabolomics of root exudates

**Figure 5:**
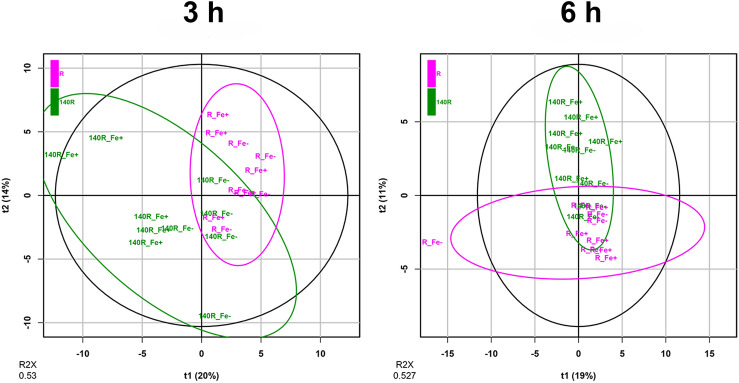
Score scatter pots of the two PCA models obtained with root exudates collected after 3 and 6 h. PCA model for samples collected at 3 h (R^2^X = 0.530, 5 PCs; scaling: mean-centering and unit variance scaling) from Fe-sufficient (Fe+) and Fe-deficient (Fe−) microcuttings of Ramsey and 140R rootstocks. PCA model for samples collected at 6 h (R^2^X = 0.527, 5 PCs; scaling: mean-centering and unit variance scaling) from Fe-sufficient (Fe+) and Fe-deficient (Fe−) microcuttings of Ramsey and 140R rootstocks.

Exudates were collected from the roots of Fe-deficient and Fe-sufficient microcuttings at 3 and 6 h using a nutrient solution (NS) with the same composition as that used for their growth, and containing either 30 μM Fe (Fe+) or no source of Fe (Fe−) (Supplementary Fig. S3). To shed some light on the roots’ response to Fe shortage in terms of exudation, we characterized exudate composition using a high-resolution untargeted mass spectrometric approach. The analyses resulted in an estimated 109 metabolites, based on the in-house root exudate database (Supplementary Table S1). Two different datasets of compounds were identified from the exudates of Ramsey and 140R rootstocks grown under Fe-sufficient and Fe-deficient conditions. The first dataset, consisting of 104 metabolites, refers to samples collected after 3 h of treatment, whilst the second one, totalling 96 compounds, refers to exudates collected after 6 h (Supplementary Table S2). Principal Component Analysis (PCA) mostly confirmed a genotype-dependent clustering of the root exudate samples both at 3 and 6 h (Fig. 5). In both datasets, the metabolite profiles of each variety were compared separately. Unsupervised PCA analyses revealed a clustering of exudate samples associated with the plant’s nutritional status (Fe-sufficiency and Fe-deficiency) for both rootstocks after 3 and 6 h (Supplementary Fig. S4, S7, S10 and S13). Supervised Orthogonal Projections to Latent Structures-Discriminant Analyses (OPLS-DA)^25^ (Supplementary Figs. S5, S6, S8, S9, S11, S12, S14, S15) were carried out in order to identify the metabolites characterizing the exudates of Fe-deficient and Fe-sufficient microcuttings. Given the large number of compounds, to simplify data interpretation the metabolites were subjected to Chemical Similarity Enrichment Analysis (ChemRICH), yielding sets of chemically similar compounds (Supplementary Fig. S16). The ChemRICH plot based on the calculated fold-change and VIP score (derived from OPLS-DA analyses, Supplementary Table S3) depicted a general decrease in the number of metabolites present in the root exudates of Fe-deficient plants as compared to that of Fe-sufficient ones. A Tanimoto chemical similarity analysis indicated several chemical clusters, with phenylpropanoids as the most prevalent compounds in both genotypes (Supplementary Table S4). The ChemRICH analysis revealed distinctive exudation patterns linked to the genotype and the sampling time. Flavonoids and lignans were well represented in both Ramsey and 140R, although the greatest alteration was observed in Ramsey. However, saturated fatty acids were the main discriminant group for the 140R rootstock. Caffeic acids displayed a slight increase at 3 h in both genotypes under Fe-deficiency conditions, whereas basic amino acids, xanthophylls and isoflavones were only evident in Ramsey, both at 3 and 6 h.

The alterations between the Fe-sufficient and Fe-deficient conditions of the compounds displaying the highest VIP values were confirmed by t-test analysis (Supplementary Table S6). In order to highlight the most substantial changes in response to Fe availability, we focused on the compounds with a percentage variation (increase or decrease) of at least 5% between Fe-sufficient and Fe-deficient conditions (Table 1). Like the morpho-physiological parameters, the metabolomic profiling of root exudates confirmed a different response to Fe availability between the two rootstocks. Ramsey microcuttings displayed 4 and 6 differentially abundant compounds between the two nutritional conditions after 3 and 6 h, respectively (Table 1). Of these, only one metabolite in the former case and two in the latter were present at higher levels in the samples of Fe-deficient rootstocks. As regards 140R, only 2 of the 16 compounds collected after 3 h, the abundance of which significantly differed in Fe-sufficient and Fe-starved microcuttings, were present at greater levels in the latter rootstocks (Table 1). For this genotype, the number of detected differences fell to 10 after 6 h, with only one metabolite increasing under Fe-deficiency conditions (Table 1).

**Table 1:** Metabolites displaying differential abundance between root exudates collected from Fe-sufficient and Fe-deficient microcuttings of Ramsey and 140R rootstocks at 3 and 6 h.

Compound	% variation	p-value	VIP
**Ramsey Fe+ vs. Ramsey Fe−; 3 h**			
*Increased abundance*			
l-glutamate	16,160.03	0.004	2.19
Cyclolariciresinol	8.19	0.0393	1.74
Caprylic acid	61.19	0.0498	1.71
*Decreased abundance*			
Campesteryl ferulate	19.36	0.0169	2
**140R Fe+ vs. 140R Fe−; 3 h**			
*Increased abundance*			
3-Sinapoylquinic acid	6.25	0.0016	1.85
p-Coumaric acid-4-*O*-glucoside	5.07	0.0022	1.80
Apigenin 6-C-glucoside	5.57	0.0042	1.75
2-Methoxy-5-prop-1-enylphenol	18.17	0.0086	1.66
Cyclolariciresinol	9.94	0.0074	1.65
Avenanthramide 2f.	5.79	0.0114	1.64
1-Acetoxypinoresinol	5.56	0.0111	1.62
Isoxanthohumol	5.31	0.0194	1.53
5-Heneicosenylresorcinol	9.74	0.0256	1.49
Caffeic acid ethyl ester	10.29	0.026	1.48
Benzoxazinone	7.63	0.0273	1.47
l-histidine	11.16	0.0458	1.40
l-ascorbate	54.86	0.04	1.38
Gallic acid ethyl ester	6.14	0.0492	1.33
*Decreased abundance*			
Campesteryl ferulate	45.32	0.0408	1.42
Schottenol ferulate	13.52	0.0381	1.41
**Ramsey Fe+ vs. Ramsey Fe−; 6 h**			
*Increased abundance*			
l-glutamate	63,740.98	< 0.0001	2.50
Maltose/sucrose	216.50	0.0043	2.00
l-tyrosine	242.13	0.0074	1.98
Homoveratric acid	52.02	0.0209	1.76
*Decreased abundance*			
Sakuranetin	60.09	0.0352	1.71
4-Hydroxycoumarin	6.93	0.0462	1.66
**140R Fe+ vs. 140R Fe−; 6 h**			
*Increased abundance*			
l-glutamate	25,186.35	< 0.0001	2.17
Caffeic acid 4-*O*-glucoside	82.92	0.0049	1.79
Maltose/sucrose	98.31	0.0047	1.77
Wighteone	15.30	0.0073	1.74
Episesamin	15.30	0.0073	1.74
3,4 Dihydroxyphenyl-2-oxypropanoic acid	35.43	0.0101	1.69
2-Methoxy-5-prop-1-enylphenol	8.36	0.0165	1.60
2-Hydroxybenzoic acid	5.37	0.0253	1.52
l-methionine	67.82	0.0429	1.46
*Decreased abundance*			
Campesteryl ferulate	11.19	0.0302	1.55

The percentage increase or decrease, p-value for t-test and VIP value from OPLS-DA are reported for each compound.

In both Ramsey and 140R exudates sampled at 3 h, the concentration of cyclolariciresinol was higher for the Fe-sufficient microcuttings, whilst campesteryl ferulate was greater in Fe-deficient ones (Table 1). The concentrations of l-glutamate and maltose/sucrose increased after 6 h in the Fe-sufficient samples of both rootstocks (Table 1).

## Discussion

140R is a well-known rootstock variety that is considered tolerant to lime-induced chlorosis^11,22^. In Ramsay, however, this trait is less clear. Galet and Smith^24^ classify it as susceptible to Fe shortage, while Bavaresco and co-workers^23^ consider it tolerant. The latter authors suggest that it may respond to lime-induced stress using a protective mechanism (e.g*.* slower growth rate and smaller root system) rather than an adaptive one, typically displayed by other tolerant rootstocks such as *Vitis berlandieri*, a parent of 140R^23^. This latter variety has been characterized not only in terms of changes in its growth parameters^14^, but also of physiological and biochemical responses to the low Fe availability^26–28^. Our experimental conditions were able to induce Fe-deficiency symptoms in both rootstocks, as confirmed by their lower leaf SPAD index (Fig. 1) and Fe content in root and shoot tissues (Fig. 3). As regards the response of Ramsey to Fe shortage, our data agree with previous findings reported in the literature^23^, despite some differences in plant material (cuttings *vs* microcuttings) and experimental conditions (calcareous soil vs. hydroponics in the absence of Fe). The SPAD indexes of the two varieties were similar for each nutritional condition (Fig. 1 and Supplementary Fig. S17), thus confirming that *Vitis berlandieri* and *Vitis champini* are among the rootstocks having the highest leaf chlorophyll content when Fe-starved^23^. Moreover, under our experimental conditions, Fe-deficient Ramsey microcuttings displayed an underdeveloped root system^23^, as emphasized by the significantly increased ratio between shoot and root length only observed in this variety (Fig. 2; Supplementary Fig. S1).

It has been reported that hybrids of *Vitis vinifera* and *Vitis berlandieri* are more responsive to Fe deficiency: several investigations performed in hydroponics^28–31^ suggest that this can be ascribed to changes in their biochemical activities, such as enhanced H^+^ extrusion and accumulation of organic acids in the roots^32^. In addition to root acidification, measuring Fe(III)-reductase activity has been proposed as a valid method to screen genotypes tolerant to Fe chlorosis^12,13^. It should be added however that some questions have been raised on the appropriateness of using the former parameter to discriminate this trait^14^. With respect to these biochemical activities, Ramsey and 140R displayed some differences in their response to Fe deficiency. Only 140R exhibited a significantly greater H^+^ extrusion from the roots of Fe-starved microcuttings than from those of Fe-replete ones (Fig. 4a), thus confirming previous data reported for this rootstock^26,28^. Moreover, root H^+^ release activity was significantly higher in 140R than Ramsey, in particular under conditions of Fe shortage (Supplementary Fig. S18). As regards FCR activity, we observed that in both rootstocks, Fe-sufficient roots displayed a greater ability to reduce Fe(III) than Fe-deficient ones (Fig. 4b). This result was rather unexpected, given that increased FCR activity is a well-established response to Fe shortage in *Strategy I* species^4^. Furthermore, the literature reports of hydroponics-grown Fe-deficient (1 μM) and Fe-sufficient (20 μM) 140R cuttings displaying enhanced FCR activity under the former conditions^26^. The fact that our results agree with those reported by Siminis et al.^27^, who used similar plant material (micropropagated 140R) and grew the plants in the absence of Fe (0 μM), stresses the importance of the presence of at least some Fe for the FCR enzyme to function^33^. Significant differences in the levels of FCR activity emerged between Fe-replete Ramsey and 140R, with the latter variety displaying the higher value (Supplementary Fig. S2). Hence, as regards the two most relevant root activities involved in *Strategy I* responses, we can conclude that Ramsey appears to be less responsive than 140R to this nutritional disorder.

In order to study in greater detail the different responses to Fe shortage between the two rootstocks, we analyzed their exudate profile. Root exudates are a mix of primary (e.g. carbohydrates, amino acids and organic acids) and secondary metabolites (e.g. flavonoids and glucosinolates)^34^ that are released into the rhizosphere, where they can interact with other plant species, microorganisms and/or abiotic components of the soil such as nutrients^35^. In our study, multivariate statistics and the subsequent ChemRICH analysis revealed a distinct metabolic signature of root exudates for Ramsey and 140R, regardless of the time-point of collection, indicating a genotype-dependent response to Fe deficiency. Nonetheless, the two genotypes also displayed some differences between the exudates harvested after 3 and 6 h. There were some similarities between the two varieties, and phenylpropanoids (mainly flavonoids, lignans and coumaric acids) were well represented in all the profiles. Our results agree with those described by Astolfi et al.^20^, showing that tomato root exudation is strongly affected by Fe deficiency, and characterized by the accumulation of amino acids, coumaric acids and other phenylpropanoids (Supplementary Figures S16). In addition, a trend similar to that described for tomato exudates was observed for saturated fatty acids and caffeic acids^20^ (Supplementary Figure S16). The lower levels of metabolites exuded by Fe-deficient plants may depend on the long period of growth in the absence of Fe (35 days). Under such prolonged stressful conditions, the plants could have switched off part of the early metabolic activities involved in the response to this disorder.

The analysis of the metabolites having a percentage variation of at least 5% between the two nutritional conditions (Table 1) indicated that 4 compounds, namely l-glutamate, cyclolariciresinol, maltose/sucrose and campesteryl ferulate, were modulated in the exudates of both rootstocks at both sampling time points. The abundance of primary metabolites, i.e. l-glutamate and maltose/sucrose, was dramatically reduced under conditions of Fe shortage. Different results have been reported for *Strategy II* species such as maize and barley, where the levels of glutamate and sugar exuded were higher in Fe-starved plants^36,37^. On the other hand, root of Fe-sufficient cucumber genotypes were observed to exude greater amounts of glutamate than Fe-deficient ones^38^. Our results suggest that the exudation of glutamate and sugars could be linked to the levels of endogenous Fe present in the grapevine plants. Other amino acids too (Table 1) appear to follow a similar trend, with a rootstock-specific response (l-tyrosine in Ramsey and l-histidine and l-methionine in 140R). This could be an adaptive response aimed at reducing the competition for Fe from rhizospheric microorganisms^39–41^. It has been reported that the decreased amounts of root exudates present at the rhizosphere as a result of the plant’s ability to reabsorb amino acids and other components along the root elongation zone, together with the release of antimicrobial metabolites, can reduce microbial competition for the Fe mobilized by root activities^42^. Both rootstocks also modulated two stress-related metabolites in response to Fe availability. In particular, we observed a reduction in lignan cyclolariciresinol^43^ and an increase in campesteryl ferulate (Table 1). It has been reported that stearyl ferulates have an antioxidant action^44,45^ and campesteryl ferulate is presumed to be involved in Fe(III) reduction, given that γ-oryzanol (a mixture of phytosteryl ferulates that also includes campesteryl ferulate) can reduce the Fe(III)/ferricyanide complex in-vitro^45^.

Other genotype-specific changes involve secondary metabolites, possibly having distinct roles in the plant’s response to Fe availability. Exudates of Fe-deficient Ramsey microcuttings (3 h) contained sakuretin^46^, an antimicrobial phytoalexin that can reduce microbial activity, thus limiting the decomposition of components involved in Fe mobilization and/or reducing the microbial competition for this nutrient^42^. In this rootstock variety we also observed a similar pattern for 4-hydroxycoumarin after 3 h (Table 1). This result is in line with the reported increase in coumarins observed in *Arabidopsis*^16–18,47^ and tomato^20^ exudates as a consequence of Fe starvation^16–18,47^. The secretion of phenolic compounds, including coumarins, involved in the chelation and/or reduction of Fe(III), is reported as an additional component of *Strategy I*^18,19^. Coumarins however can differ in their power to mobilize Fe(III) from Fe hydroxide^18^, and their ability to chelate and reduce Fe(III) in vitro^48^: in this respect, 4-hydroxycoumarin is considered a rather poor Fe(III)-chelating agent^48^. This compound however, present in Ramsey exudates, is toxic for soil bacteria, and its release into the rhizosphere would consequently increase the competitive edge of grapevine roots for Fe, as already proposed for *Arabidopsis*^16,19^. Moreover, 4-hydroxycoumarin is reported to have allelopathic properties^49^.

As regards 140R-specific modulations, Fe-deficiency caused a decrease in the metabolites or derivatives commonly found in plant exudates, such as salicylic acid (2-hydroxybenzoic acid), p-coumaric acid (p-coumaric acid-4-*O*-glucoside), some caffeic acids (caffeic acid ethyl ester and caffeic acid 4-*O*-glucoside) and flavonoids (apigenin 6-C-glucoside, isoxanthohumol and wighteone)^34,50^. Several of these compounds are believed to play a role in plant defence responses at the rhizosphere thanks to their antibacterial (apigenin 6-C-glucoside)^51^, antifungal (wighteone)^52^ or oestrogenic (isoxanthohumol) action^53^. In addition, other two compounds whose levels decrease in response to Fe-deficiency, namely avenanthramide f^54^ and benzoxazionone^55^, may be involved in the interaction with other plants and microorganisms. Exudates of 140R Fe-deficient microcuttings contained lower levels of lignans such as 1-acetoxypinoresinol^56^ and episesamin^57^. Lignans too can exhibit insecticidal, oestrogenic, antiviral, antibacterial and antioxidant properties^58^. Unlike 140R, Fe-deficient *Arabidopsis* roots have been reported to release greater amounts of non-conventional lignans, i.e. coumarinolignans^59^. These compounds are assumed to play two complementary roles for the acquisition of Fe, namely acting as Fe(III) mobilizers and allelochemicals^59^. We observed a general downward trend in the release of phenolics under Fe deficiency conditions. We cannot discard the hypothesis that in 140R, the prolonged absence of any source of Fe may have switched off at least some mechanisms involved in the response to Fe shortage. Furthermore, exudates of Fe-deficient 140R microcuttings contained lower levels of an antioxidative metabolite, l-ascorbate^60^. Ascorbate is a strong reducing agent for Fe^3+^ and could be involved in the reduction and acquisition of Fe from ferric complexes as described in pea embryos^61,62^. This strategy of Fe acquisition was not induced by Fe-deficiency, unlike root ferric chelate reductase activities^62^, and this would explain the higher concentrations of this metabolite observed under Fe-sufficient conditions.

With regard to other mechanisms involved in Fe acquisition from the rhizosphere, we detected phytosiderophore 3-hydroxymugineic acid^63^ in the exudates of both rootstocks (Supplementary Tables S1, S2). Its release into the soil is considered a typical response of Fe-deficient *Strategy II* species^2^, but our data indicate that its concentration was not increased by Fe-deficiency (Supplementary Table S6). It has been recently suggested that 3-hydroxymugineic acid could play a role in Fe acquisition by Fe-sufficient tomato plants^20^. These results seem to indicate that the secretion of phytosiderophores as a function of the plant’s Fe status is similar in both herbaceous and woody *Strategy I* plants. Interestingly, the phytosiderophore 2′-deoxymugineic acid was identified in the leaves of another tree species, olive, at levels seemingly independent of its Fe status^64^.

**Figure 6:**
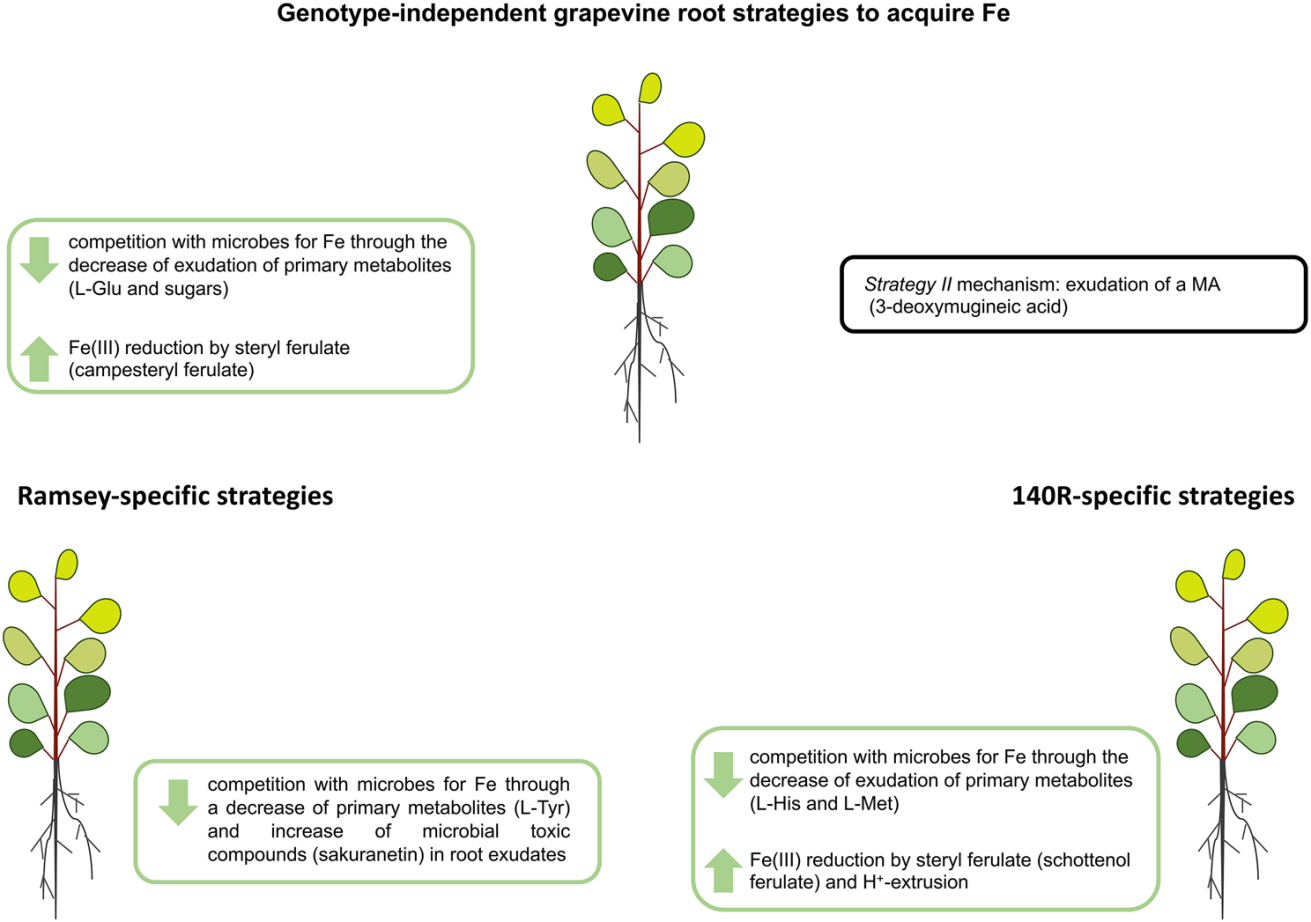
Strategies adopted by grapevine roots to acquire Fe. The genotype-independent, Ramsey-specific and 140R-specific strategies are reported in the schematic. In the green boxes: strategies affected by Fe-deficiency; in the black box: strategies independent of Fe-deficiency. Glu: glutamate; L-Tyr: l-tyrosine; MA: mugineic acid; L-His: l-histidine; L-Met: l-methionine.

In conclusion, these two grapevine rootstocks appear to stimulate Fe acquisition by reducing the microbial competition for this micronutrient and increasing the reduction of Fe(III) by stearyl ferulate (Fig. 6). Interestingly, our analyses of exudate composition revealed that the two rootstocks could also release MA (3-deoxymugineic acid), a mechanism usually associated with *Strategy II* species, although in our case the compound was extruded independently of the rootstock’s Fe status (Fig. 6). It remains to be clarified whether the amount extruded is significant in the overall context of Fe acquisition and/or if the possibly chelated Fe(III) is used after reduction^65^ or through components of the *Strategy II* mechanism such as TOM- and YS1-like proteins.

Some strategies for Fe acquisition were observed to be genotype-dependent (e.g*.* the reduction of certain amino acids), thus confirming pioneering works^66^ showing a genetically-based variability in the plants’ responses to Fe deficiency (Fig. 6). 140R in particular is more responsive to Fe deficiency as suggested by its substantial modulation of biochemical reactions typical of *Strategy I* species. In fact, this rootstock exhibits a greater ability to increase Fe availability at the rhizosphere as suggested by the increased H^+^ extrusion and the secretion of larger amounts of ferulate conjugates (e.g. schottenol ferulate), which can play a role in Fe(III) reduction (Fig. 6). Ramsey on the other hand, despite displaying more limited growth in response to Fe deficiency and an inability to boost H^+^ extrusion, was observed to specifically increase its competitive edge over soil microbes by releasing toxic compounds such as sakuranetin from its roots (Fig. 6).

Taken together, our data underline that, even within the same genus, the mechanisms involved in the response to Fe shortage are very diverse, and the response itself cannot be simply described in terms of variations in the activity of the components usually associated with *Strategies I* and *II*.

## Methods

### Plant growth

Microcuttings of two grapevine rootstocks, Ramsey and 140 Ruggeri (140R), were hydroponically grown for 7 days in plastic pots containing 2.2 L of 0.5 mM CaSO_4_ (6 microcuttings per pot) at 200 μmol m^−2^ s^−1^ PPFD with a 16 h/8 h day/night regime at 24 °C and 80% relative humidity. The microcuttings of both rootstock varieties were then divided into two groups and grown hydroponically for 28 days in a nutrient solution (NS) containing either 30 μM FeNaEDTA (Fe+) for the group of Fe-sufficient plants (control samples) or no source of Fe (Fe−) for the group of Fe-deficient plants (treated samples). The NS had the following composition: 0.5 mM MgSO_4_, 0.7 mM K_2_SO_4_, 2 mM Ca(NO_3_)_2_, 0.1 mM KH_2_PO_4_, 0.1 mM KCl, 10 μM H_3_BO_3_, 0.5 μM MnSO_4_, 0.5 μM ZnSO_4_, 0.2 μM CuSO_4_ and 0.01 μM (NH_4_)_6_Mo_7_O_24_. The solution was replaced every week. At the end of the growth period (35 days, 7 days of growth in CaSO_4_ plus 28 days in a NS with or without Fe), the fresh weight and length of shoot and roots were measured in each plant. The SPAD index value was determined as an average of the values measured in all leaves. The SPAD index of each leaf was calculated as the average of five measurements taken with a SPAD-502 Plus Chlorophyllmeter (Konica Minolta). Growth parameters and SPAD indexes were determined in 4 plants from three independent experiments. The mean value of growth and SPAD parameters was calculated for each independent experiment. Root and shoot tissues of 6 plants were sampled and dried at 60 °C for 48 h in order to determine Fe content by ICP-MS analysis. Other Fe+ and Fe− plants were used to determine the H^+^ release and ferric-chelate reduction activities in their roots, as well as to harvest their root exudates.

### Determination of Fe content by ICP-MS analysis

Root and shoot tissues were dried at 60 °C for 48 h. About 10 mg were digested in a 3-mL TFM microsampling insert (Milestone Srl) using 250 μL of ultrapure grade HNO_3_ (Romil). Three inserts were placed inside a TFM 100-mL vessel with 11 mL Milli-Q water and 1 mL of ultrapure grade H_2_O_2_ (69%. Fisher Scientific). The digestion was performed at 180 °C for 20 min in a StartD (Milestone Srl) microwave digestor. The digested samples were diluted to 2% HNO_3_ with ultra-pure grade water (18.2 MΩ·cm at 25 °C), and then analysed using an Agilent 7500ce ICP-MS detection system (Agilent Technologies). A calibration curve was achieved using 13 different concentrations (2–2048 μg  L^−1^) obtained by diluting a Fe standard solution (10 mg L^−1^, Romil). Measurement accuracy and matrix effect errors were checked using NIST standard reference material 1515 (Apple leaves), which was digested and analysed in the same way as the samples.

### Determination of root H^+^ release

The assay was carried out as described by Pinton et al.^67^. The roots were rinsed with deionized water (18.2 MΩ·cm at 25 °C) to remove all traces of NS. The apical roots were then cut and used for the assay, which was performed at 28 °C using roughly 0.8 g of root sample in 38 mL of an aerated solution having the following composition: 0.1 mM BTP, 3 mM KCl, pH 6.3. The pH values were determined using a Crison Basic 20 (Crison Instruments) pH-meter after 0, 5, 15, 30 and 60 min of root incubation. Data are expressed as μmol H^+^ gFW^−1^. The concentration of H^+^ was determined at each time point using the corresponding pH value. The H^+^ concentration of the solution at 5, 15, 30 and 60 min minus that at 0 min was calculated, multiplied by the assay volume and divided by the fresh weight in grams (gFW) of each root sample.

### Ferric-chelate reduction assay

The roots’ ability to reduce Fe(III)-EDTA was determined as described by Pinton et al.^68^. The roots were rinsed with deionized water (18.2 MΩ cm at 25 °C), incubated for 5 min at room temperature in 100 mL of a 0.1 mM EDTA solution and then rinses three more times with deionized water (18.2 MΩ cm at 25 °C). The root system was incubated in the dark and at room temperature in 10 mL of an assay solution having the following composition: 5 mM CaSO_4_, 10 mM MES-KOH pH 5.5, 250 μM Fe-EDTA, 500 μM BPDS. The absorbance of the solutions was measured after 30, 60, 90 and 120 min at 535 nm and the amount of reduced Fe was deduced from the concentration of the Fe(II)-BPDS_3_ complex thus formed using an extinction coefficient equal to 22.1 mM cm^−1^.

### Collection of root exudates

Microcuttings of Ramsey and 140R were hydroponically grown as previously described. After 28 days of growth in the presence or absence of Fe, root exudates of each plant were collected in a plate using 10 mL of NS having the same composition as that used throughout their growth period, hence NS with 30 μM FeNaEDTA for the Fe+ microcuttings and NS with no source of Fe for the Fe-deficient ones. Five replicates were produced by harvesting separately the exudates of 5 different plants per each genotype and condition. One millilitre of solution was collected from each plate after 3 and 6 h. The plates were closed during the experiments to avoid evaporation; roots were shielded from the light and the plates were incubated at 24 °C and 80% relative humidity.

### Untargeted metabolomics applied to root exudates

Root exudates were filtered through a 0.22 um cellulose syringe filter and metabolic profiling was carried out by UHPLC-ESI/QTOF mass spectrometry as described by Lucini et al.^69^. Chromatographic separation was achieved using water and CH_3_CN as the mobile phases (both LCMS grade, VWR, Milan, Italy). The elution gradient went from 5 to 95% CH_3_CN within 33 min, and flow rate was 220 μL min^−1^. The mass spectrometer worked in positive polarity and scan mode (100–1200 m/z).

Raw spectral data were processed using the Profinder B.06 software (Agilent Technologies) and compared to an in-house database specific for plant root exudates^20,69^. The find-by-formula algorithm was used to annotate molecular features, following mass and retention time alignment, using monoisotopic accurate mass, isotope spacing and isotope ratio. The minimum absolute abundance was set to 5000 counts (a signal-to-noise of about 5) and the mass accuracy tolerance was < 5 ppm. The root exudate metabolic profiling provided 109 annotated compounds identified as exudate samples. The whole dataset is reported as supplementary material together with the composite mass spectra (Supplementary Table S1). Two distinct datasets were obtained with the samples collected at 3 and 6 h. In both cases, the dataset consists of compounds that are present in at least one sample (104 for the 3 h dataset and 96 for the 6 h one). These data are reported in Supplementary Table S2. In both datasets, missing values were replaced by half the minimum of the non-missing values^70^ and data were log_10_ transformed. PCA and OPLS-DA analysis were performed using the “ropls” package^70,71^ for R software^72^. Chemical Similarity Enrichment Analysis (ChemRICH) was carried out by means of the online web-app tool (https://chemrich.fiehnlab.ucdavis.edu) and then used for interpretation as described by Barupal and Fiehn^73^. The metabolites were grouped into non-overlapping chemical sets using the Tanimoto substructure chemical similarity coefficients. Significance values were obtained by self-contained Kolmogorov–Smirnov tests, on the ground of their VIP score values. The multivariate analyses were carried out by applying mean-centering and unit variance scaling^70^. The t-test analysis (two-tailed, 95% confidence level) was carried out using Prism-GraphPad software to confirm the differential abundance of the variables displaying the highest VIP value.

## Supplementary information

Supplementary Table Legends.

Supplementary Information 1.

Supplementary Table S1.

Supplementary Table S2.

Supplementary Table S3.

Supplementary Table S4.

Supplementary Table S5.

Supplementary Table S6.

## Data Availability

All the data generated or analysed in this study are included in this published article (and its Supplementary Information files).
