# Supplementary material for: Changes in physiological activities and root exudation profile of two grapevine rootstocks reveal common and specific strategies for Fe acquisition
Source: Sci Rep. 2020 Nov 2;10:18839. doi: 10.1038/s41598-020-75317-w (PMC7606434; doi:10.1038/s41598-020-75317-w)
Supplement: Supplementary file 1 — Supplementary Table Legends. [file 41598_2020_75317_MOESM1_ESM.docx]

**Supplementary Information**

Supplementary Fig. S1-S18.

Supplementary Table S1. The entire metabolome dataset with retention times and composite mass spectra.

Supplementary Table S2. The two datasets of compounds identified from the exudates sampled at 3 and 6 h.

Supplementary Table S3. VIP values of each compounds for the 4 OPLS-DA models.

Supplementary Table S4. Results of Chemical Similarity Enrichment Analysis carried out in Ramsey at 3 h, 140R at 3 h, Ramsey at 6 h and 140R at 6 h.

Supplementary Table S5. Metabolites that appeared more or less abundant in the root exudates, and were used for ChemRICH analysis. VIP discriminant metabolites from OPLS-DA modelling were used to cluster metabolites into chemical groups, and Kolmogorov–Smirnov test based on VIP scores to indicate the level of significance of the clusters.

Supplementary Table S6. Differentially abundant compounds identified by comparing the metabolomic profiles of root exudates (Fe+ *vs* Fe‑, Ramsey, 3 h; Fe+ *vs* Fe‑, 140R, 3 h; Fe+ *vs* Fe‑, Ramsey, 6 h; Fe+ *vs* Fe‑, 140R, 6 h). The percentage increase or decrease, p-value for t-test and VIP value from OPLS-DA are given for each compound.
