# Supplementary material for: Changes in physiological activities and root exudation profile of two grapevine rootstocks reveal common and specific strategies for Fe acquisition
Source: Sci Rep. 2020 Nov 2;10:18839. doi: 10.1038/s41598-020-75317-w (PMC7606434; doi:10.1038/s41598-020-75317-w)
Supplement: Supplementary file 2 — Supplementary Information 1. [file 41598_2020_75317_MOESM2_ESM.pdf]

Changes in physiological activities and root exudation profile of two grapevine rootstocks reveal common and specific strategies for Fe acquisition

Laura Marastoni[1a], Luigi Lucini[2a], Begoña Miras-Moreno[2], Marco Trevisan[2], Davide Sega[1], Anita Zamboni[1*], Zeno Varanini[1]

[1]Biotechnology Department, University of Verona, Italy

[2]Department for Sustainable Food Process, University Cattolica del Sacro Cuore, Piacenza, Italy

Supplementary Information

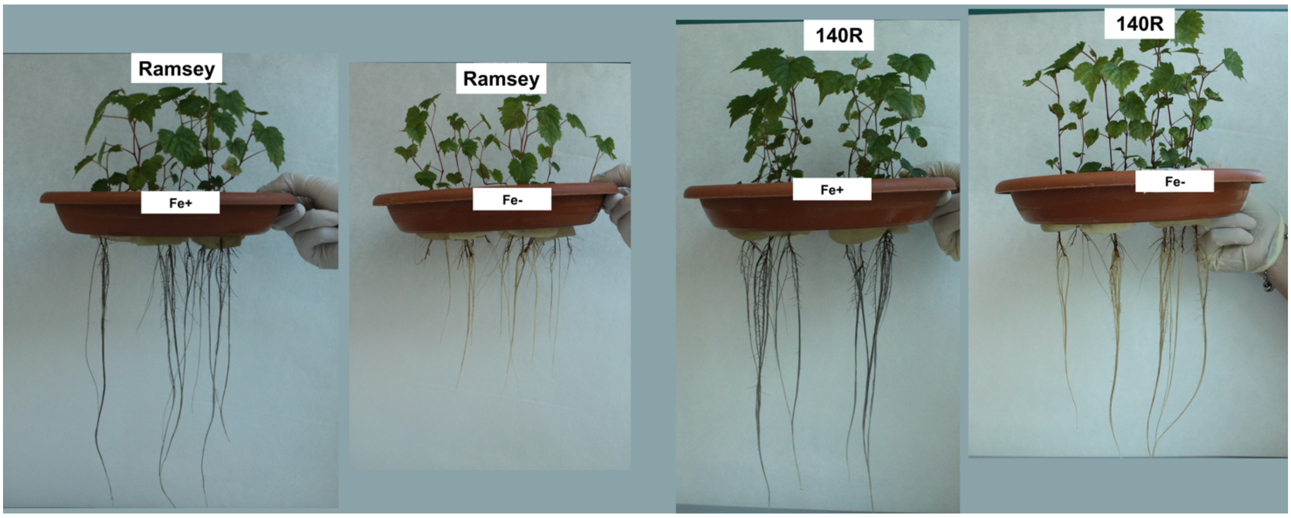

Supplementary Fig. S1. Microcuttings of Ramsey and 140R at the end of experiments grown in presence (Fe+) and absence of Fe (Fe-).

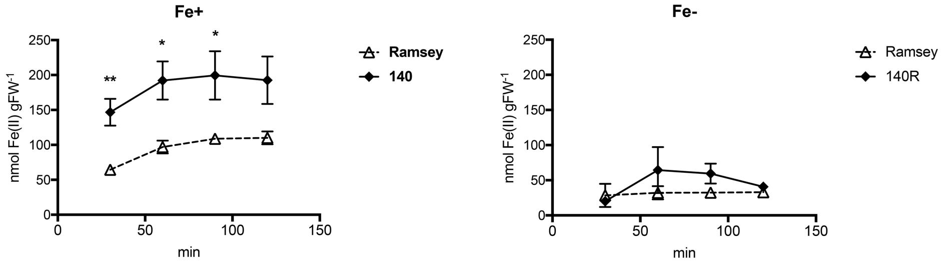

Supplementary Fig. S2. Time-course of root FCR activity. Comparison of reduction of Fe(III)-EDTA activity by root apparatus measured after 30, 60, 90 and 120 min and expressed as nmol Fe(II) gFW^{-1} between Ramsey and 140R microcuttings grown under Fe-sufficient (Fe+) and Fe-deficient (Fe-) condition. The statistical significance was determined by Student's t-test (mean ± SE, n= 4 plants, * $P < 0.05$. **, $P < 0.01$).

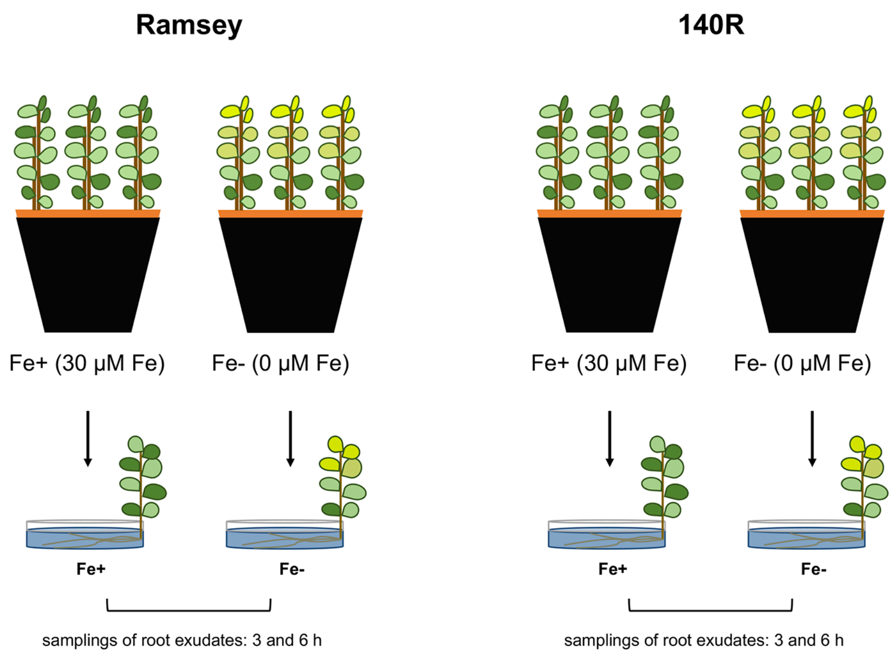

Supplementary Fig. S3. Scheme of root exudate collection. Metabolite dataset was obtained through UPLC/Q-TOF analysis of root exudates sampled after 3 h and 6 h from microcuttings of Ramsey and 140R grown with Fe or without Fe.

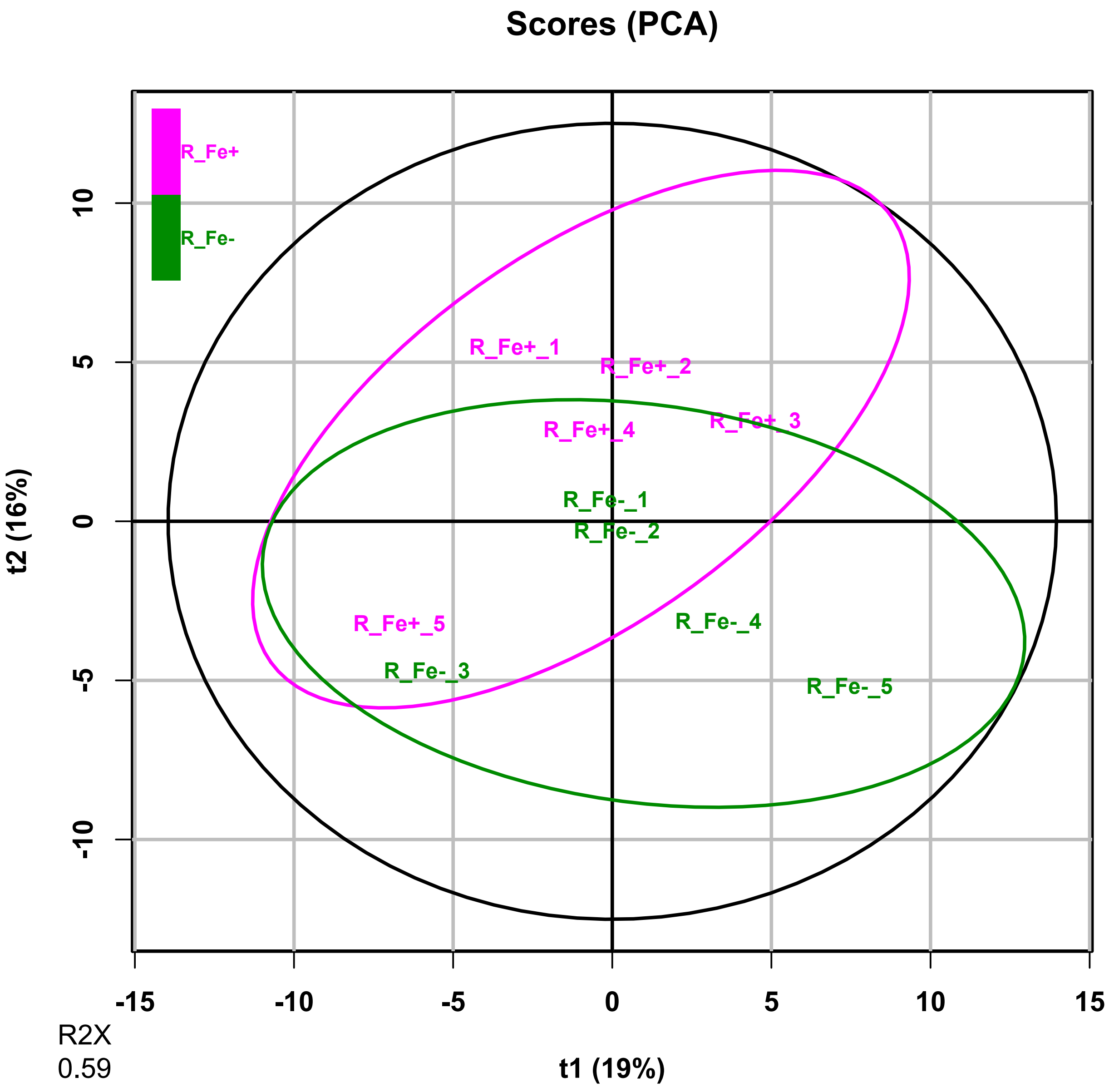

Supplementary Fig. S4. Score scatter plot of PCA model of root exudate samples of Ramsey rootstocks sampled at 3 h. PCA model (R^2X= 0.59, 4 PCs; scaling: mean-centering and unit variance scaling) of root exudates collected at 3 h from Fe-deficient and Fe-sufficient Ramsey microcuttings.

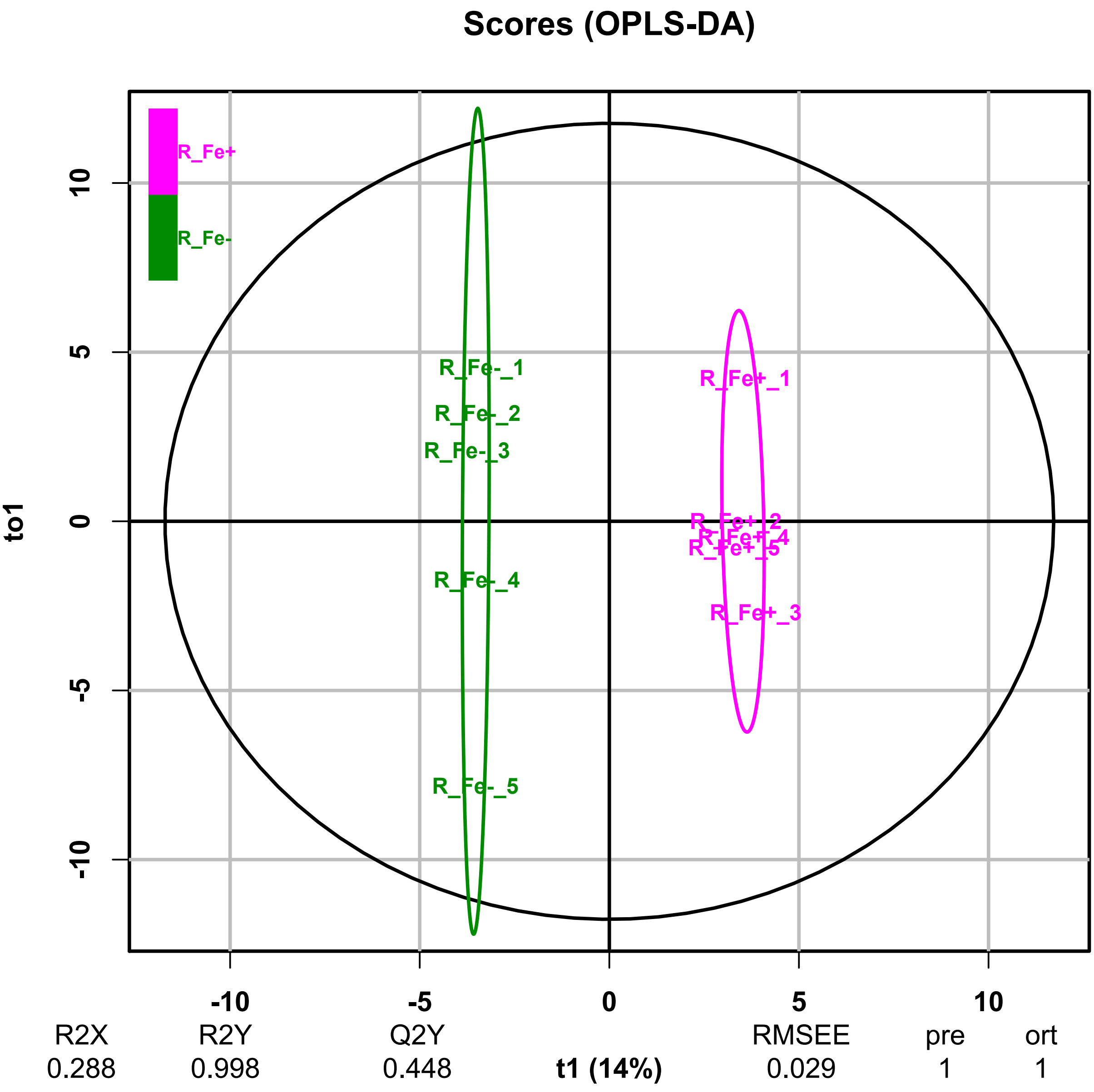

Supplementary Fig. S5. Score scatter plot of OPLS-DA model of root exudate samples of Ramsey rootstocks sampled at 3 h. OPLS-DA model (1P+1O, R^2X(cum)= 0.288, R^2Y(cum)= 0.998, Q^2(cum)= 0.448, RMSEE= 0.029; scaling: mean-centering and unit variance scaling) of root exudates collected at 3 h from Fe-deficient and Fe-sufficient Ramsey microcuttings.

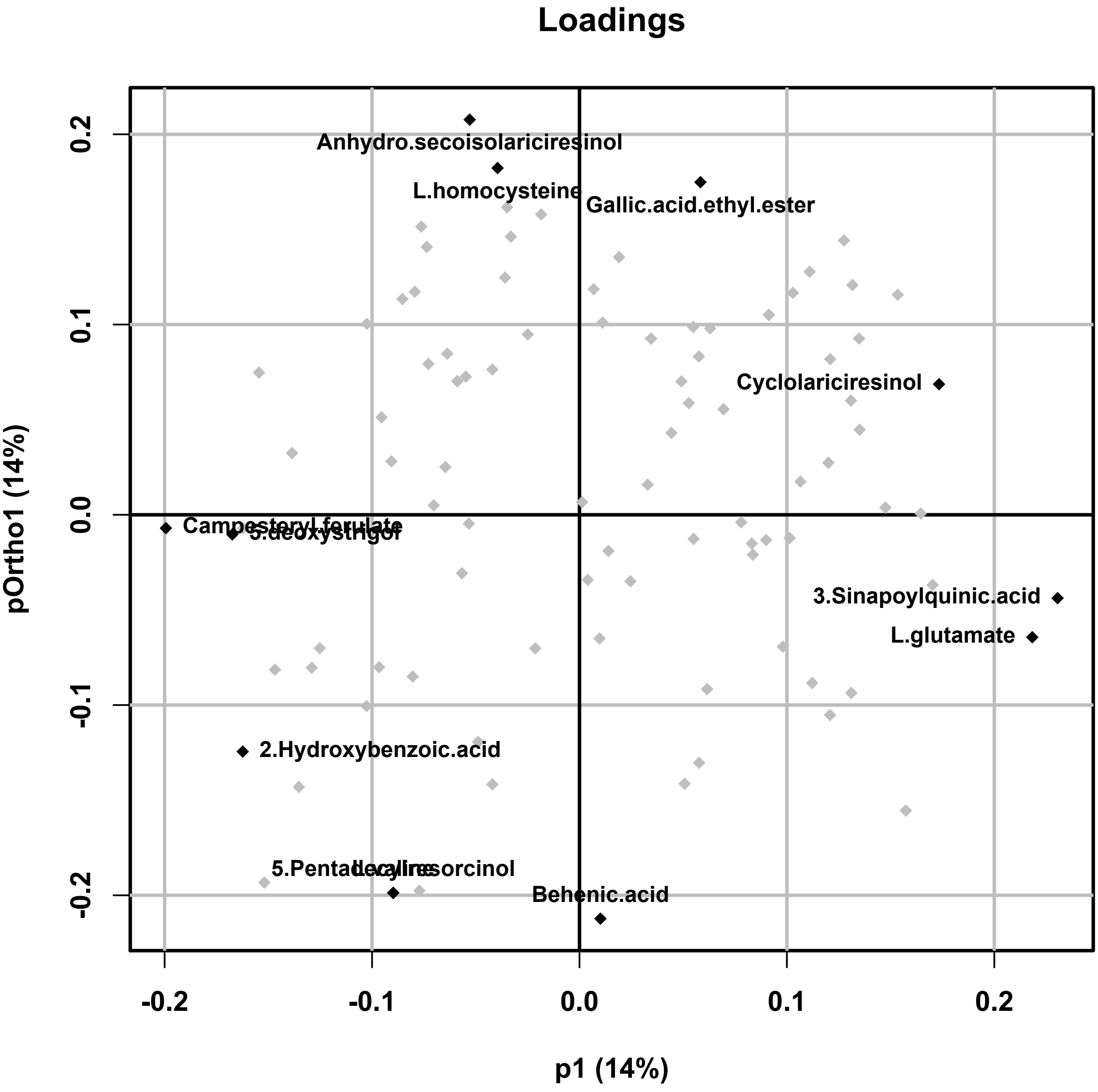

Supplementary Fig. S6. Loading plot of OPLS-DA model of root exudate samples of Ramsey rootstocks sampled at 3 h. OPLS-DA model (1P+1O, R^2X(cum)= 0.288, R^2Y(cum)= 0.998, Q^2(cum)= 0.448, RMSEE= 0.029; scaling: mean-centering and unit variance scaling) of root exudates collected at 3 h from root exudates of Fe-deficient and Fe-sufficient Ramsey microcuttings.

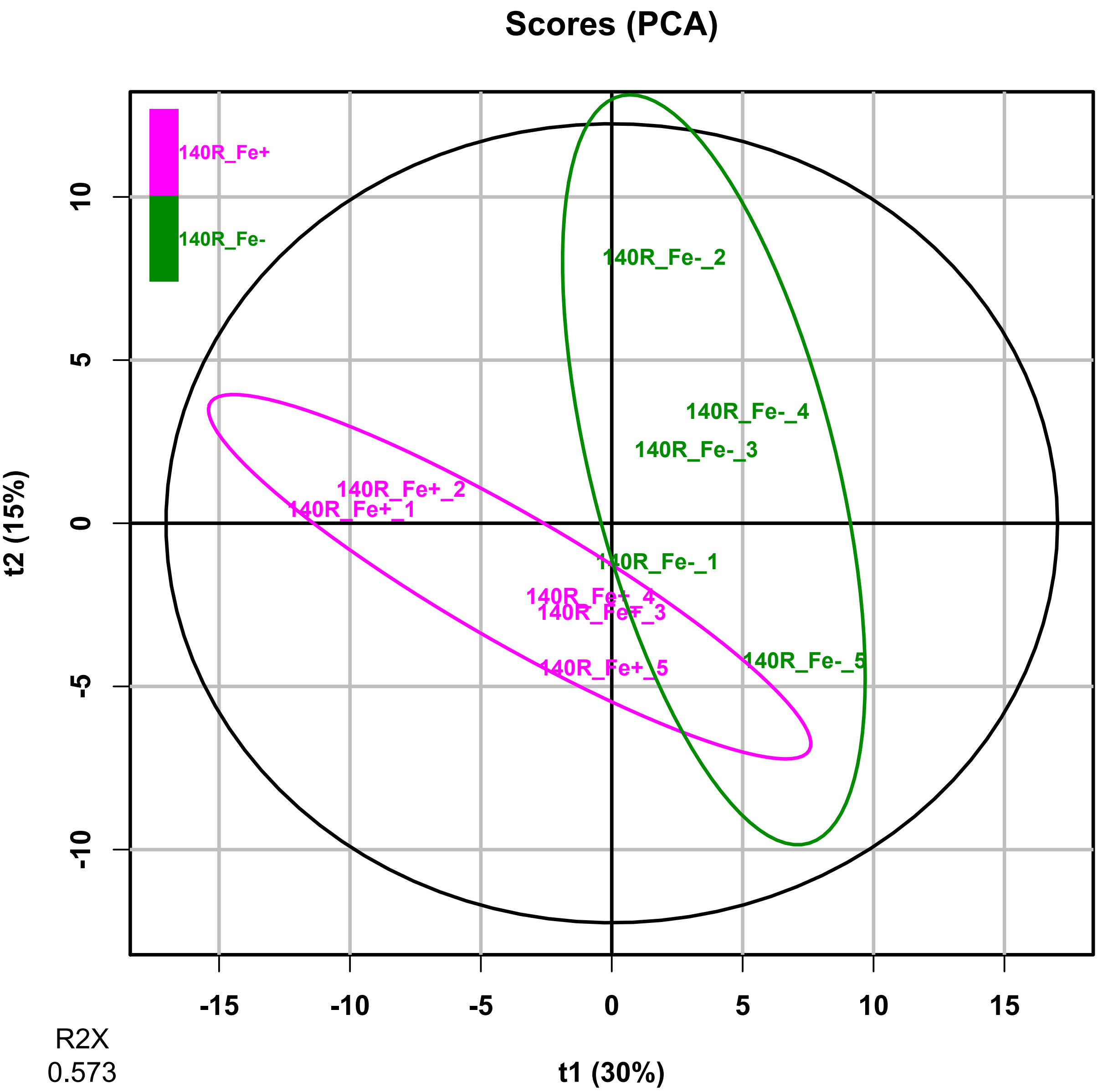

Supplementary Fig. S7. Score scatter plot of PCA model of root exudate samples of 140R rootstocks sampled at 3 h. PCA model ($R^2X_{(cum)}$= 0.573, 3 PCs; scaling: mean-centering and unit variance scaling) of root exudates collected at 3 h from Fe-deficient and Fe-sufficient 140R microcuttings.

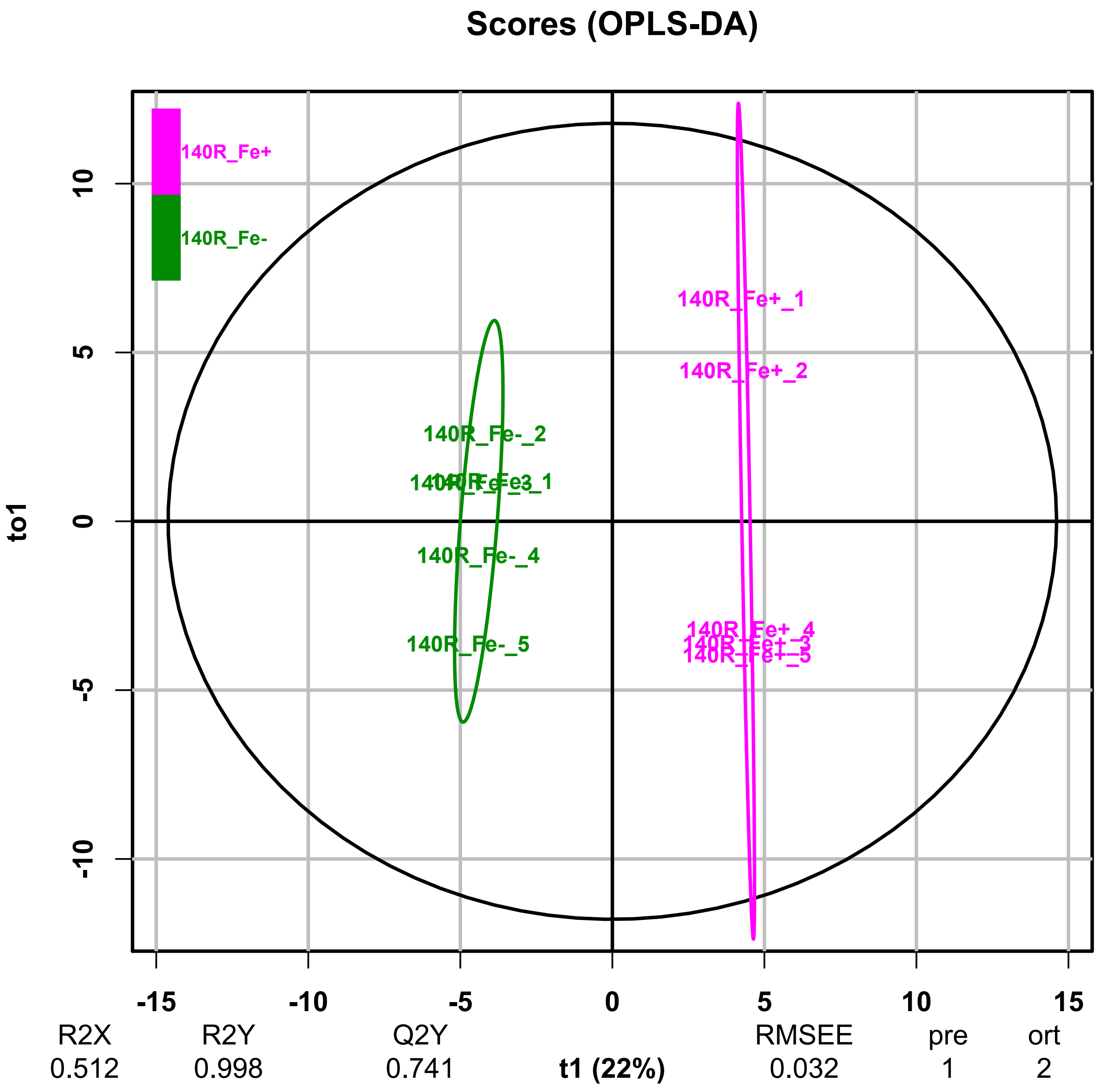

Supplementary Fig. S8. Score scatter plot of OPLS-DA model of root exudate samples of 140R rootstocks sampled at 3 h. OPLS-DA model (1P+2O, R^2X(cum)= 0.512, R^2Y(cum)= 0.998, Q^2(cum)= 0.741, RMSEE= 0.032; scaling: mean-centering and unit variance scaling) of root exudates collected at 3 h from Fe-deficient and Fe-sufficient 140R microcuttings.

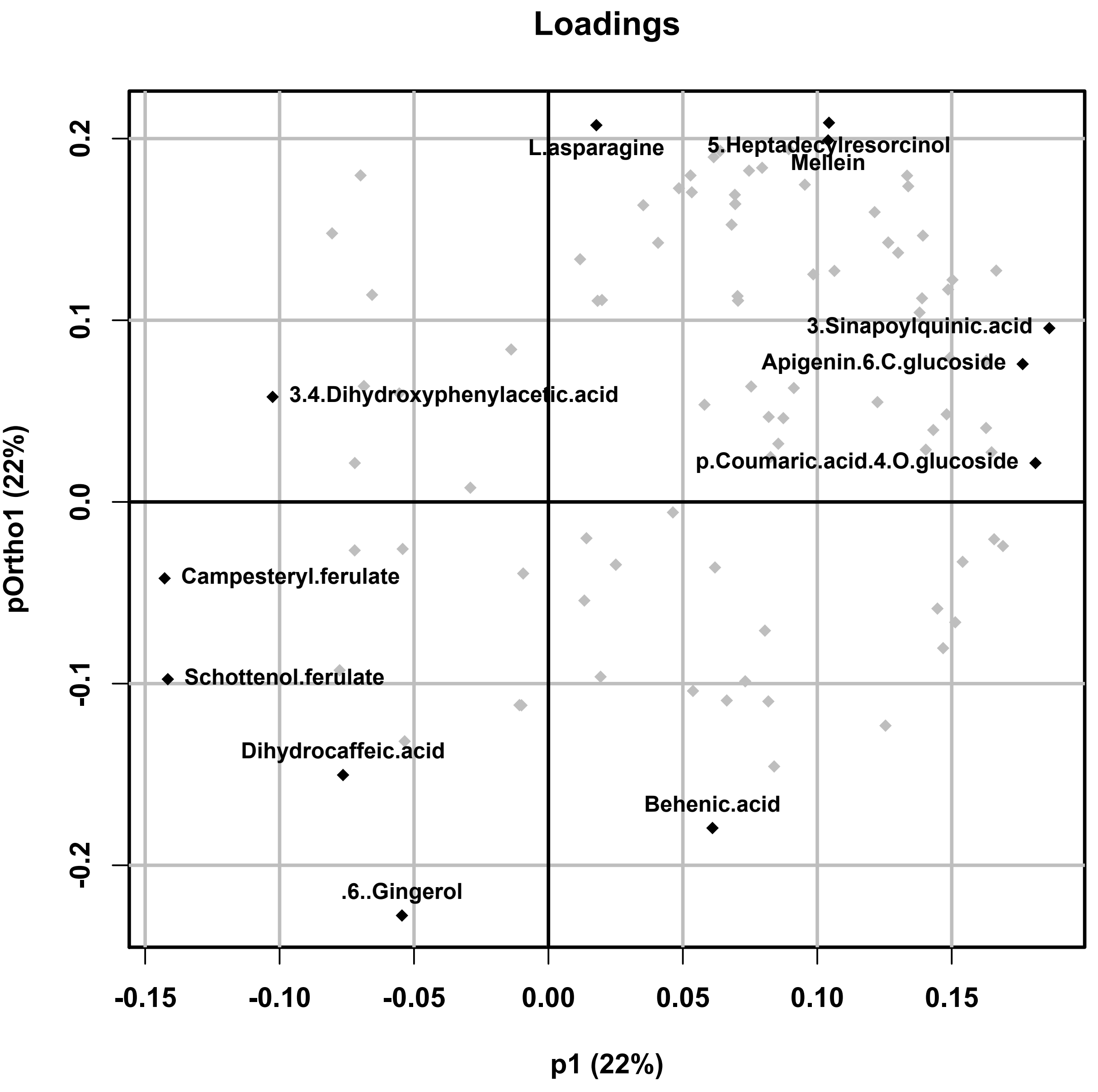

Supplementary Fig. S9. OPLS-DA model of root exudate samples of 140R rootstocks sampled at 3 h. OPLS-DA model (1P+2O, R^2X(cum)= 0.512, R^2Y(cum)= 0.998, Q^2(cum)= 0.741, RMSEE= 0.032; scaling: mean-centering and unit variance scaling) of root exudates collected at 3 h from Fe-deficient and Fe-sufficient 140R microcuttings.

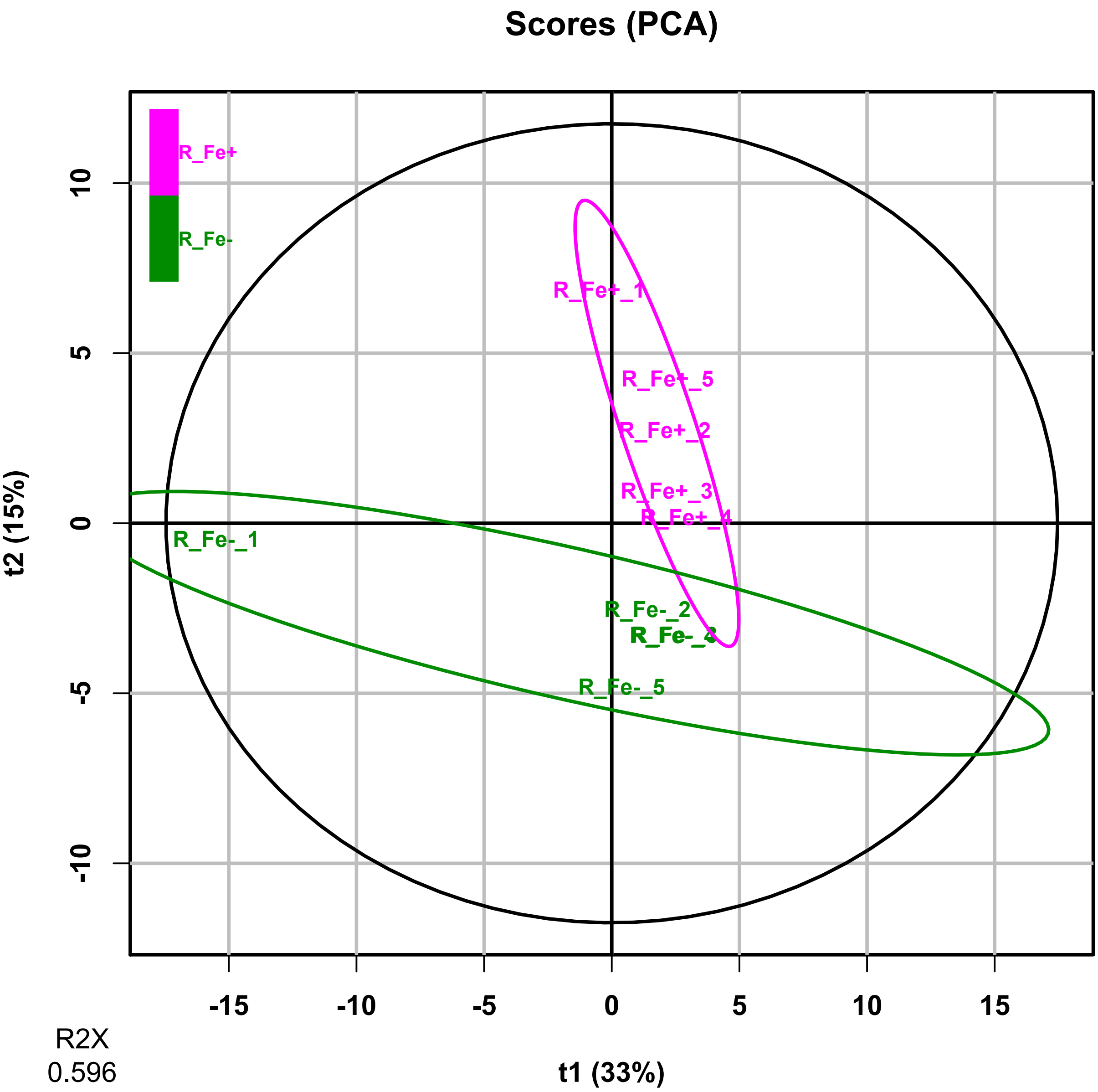

Supplementary Fig. S10. PCA model of root exudate samples of Ramsey rootstocks sampled at 6 h. PCA model ($R^2X_{(cum)}$= 0.596, 3 PCs; scaling: mean-centering and unit variance scaling) of root exudates collected at 6 h from Fe-deficient and Fe-sufficient Ramsey microcuttings.

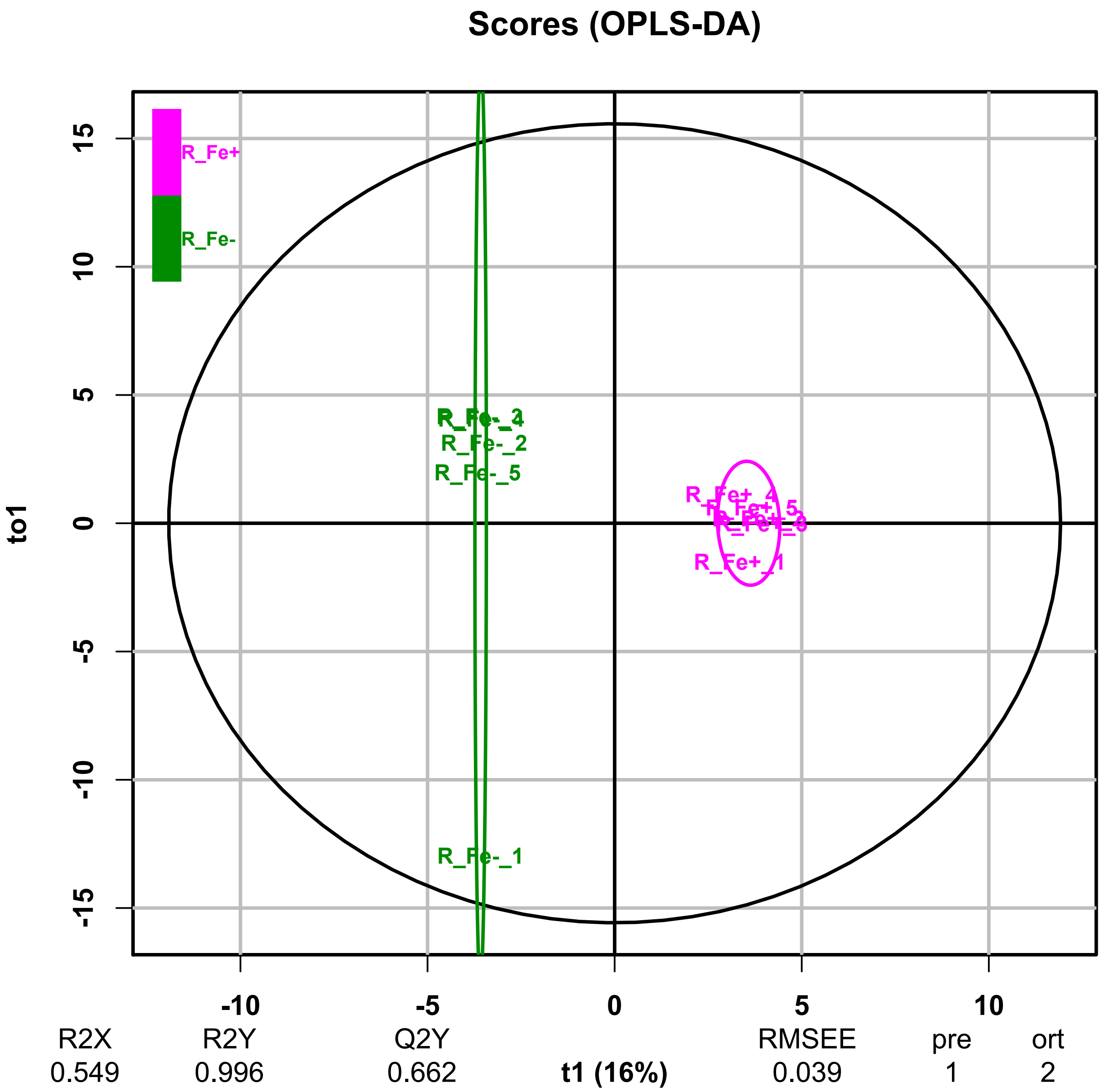

Supplementary Fig. S11. OPLS-DA model of root exudate samples of Ramsey rootstocks sampled at 6 h. Scatter plot of OPLS-DA model (1P+2O, R^2X(cum)= 0.549, R^2Y(cum)= 0.996, Q^2(cum)= 0.662, RMSEE= 0.039; scaling: mean-centering and unit variance scaling) of root exudates collected at 6 h from Fe-deficient and Fe-sufficient Ramsey microcuttings.

.

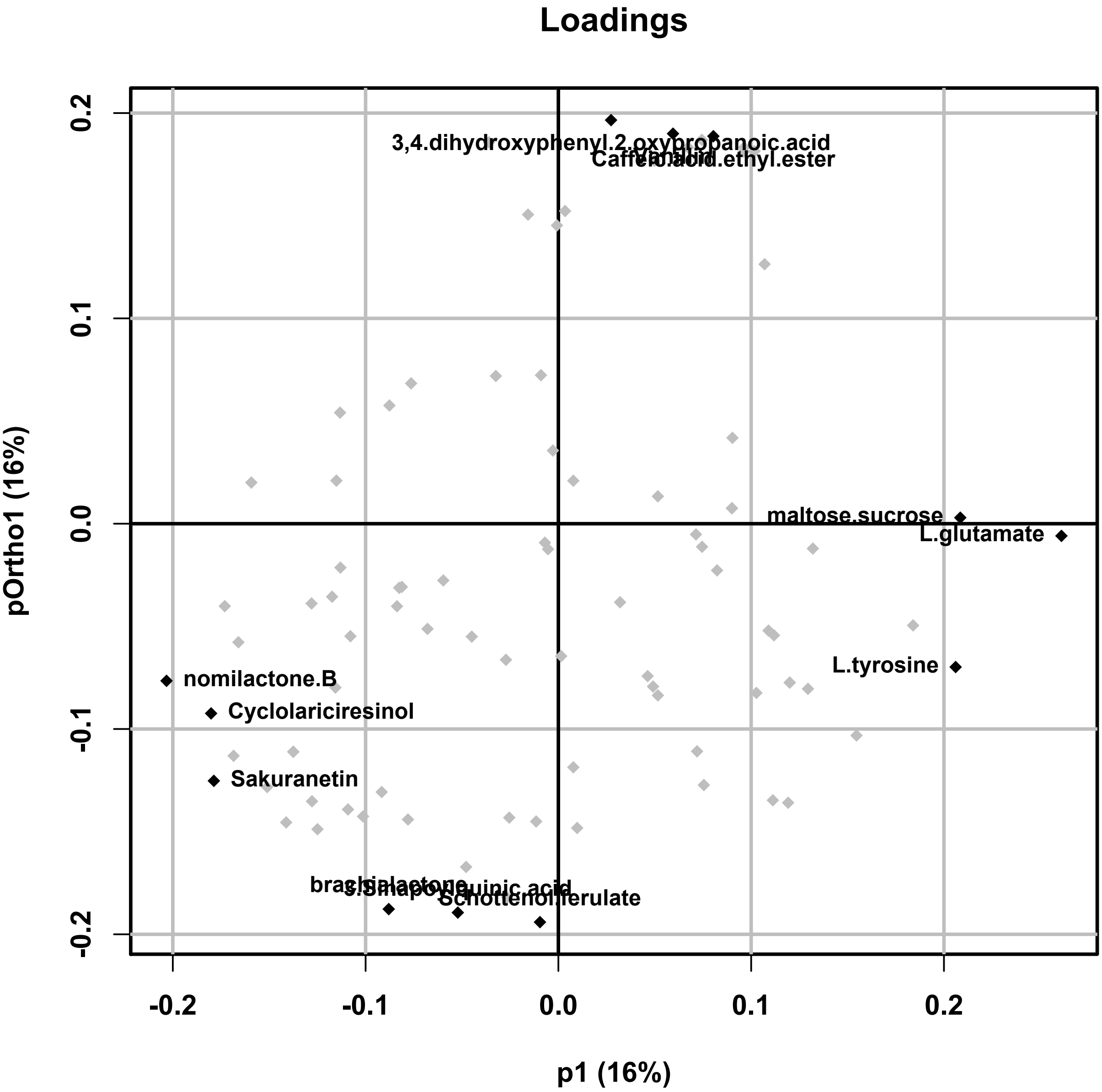

Supplementary Fig. S12. OPLS-DA model of root exudate samples of Ramsey rootstocks sampled at 6 h. Loading plot of OPLS-DA model (1P+2O, R^2X(cum)= 0.549, R^2Y(cum)= 0.996, Q^2(cum)= 0.662, RMSEE= 0.039; scaling: mean-centering and unit variance scaling) of root exudates collected at 6 h from Fe-deficient and Fe-sufficient Ramsey microcuttings.

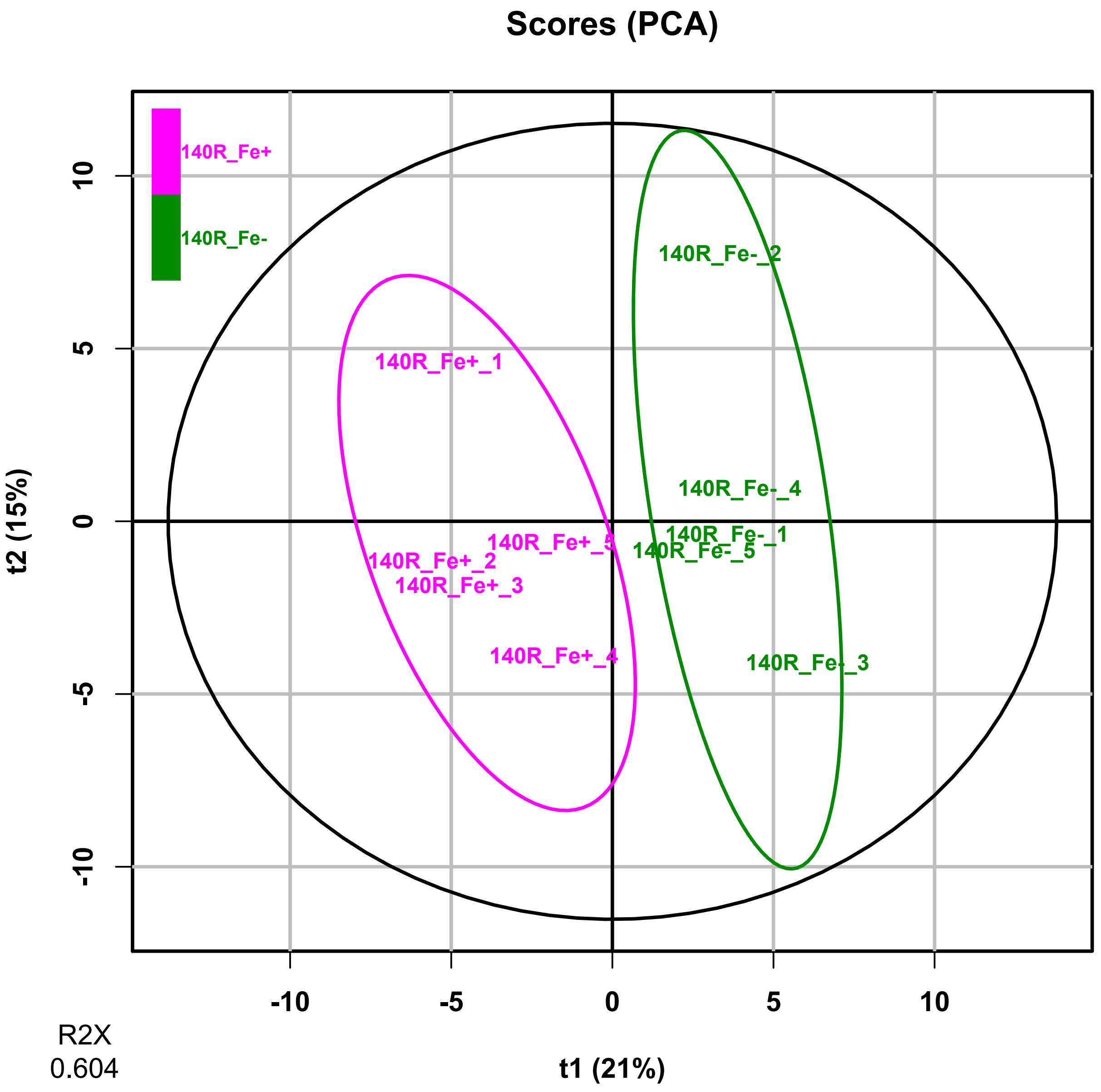

Supplementary Fig. S13. PCA model of root exudate samples of 140R rootstocks sampled at 6 h. Scatter plot of PCA model ($R^2X_{(cum)}$= 0.604, 4 PCs; scaling: mean-centering and unit variance scaling) of root exudates collected at 6 h from Fe-deficient and Fe-sufficient 140R microcuttings.

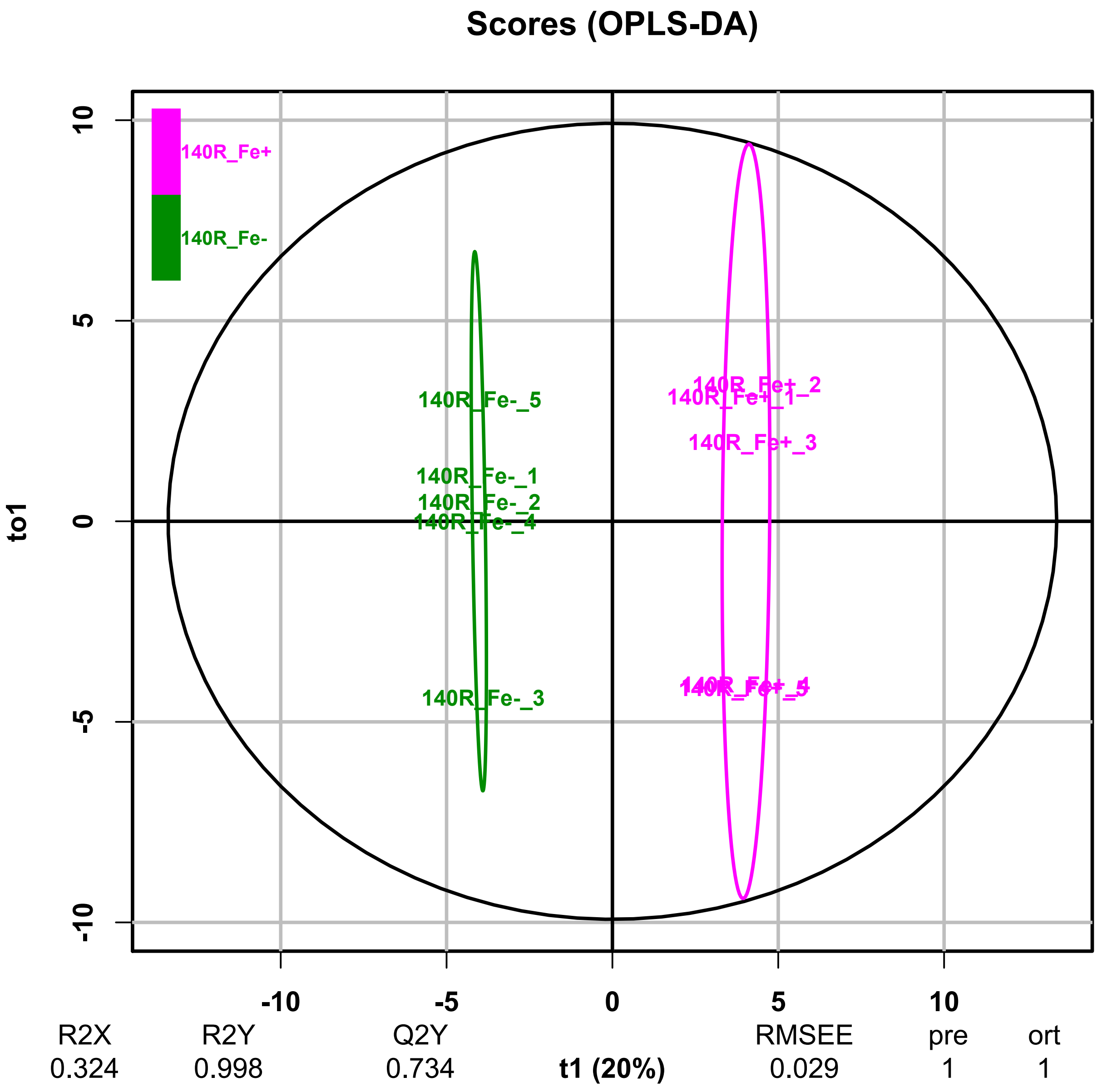

Supplementary Fig. S14. OPLS-DA model of root exudate samples of 140R rootstocks sampled at 6 h. Scatter plot of OPLS-DA model (1P+1O, R^2X(cum)= 0.324, R^2Y(cum)= 0.998, Q^2(cum)= 0.734, RMSEE= 0.029; scaling: mean-centering and unit variance scaling) of root exudates collected at 6 h from Fe-deficient and Fe-sufficient 140R microcuttings.

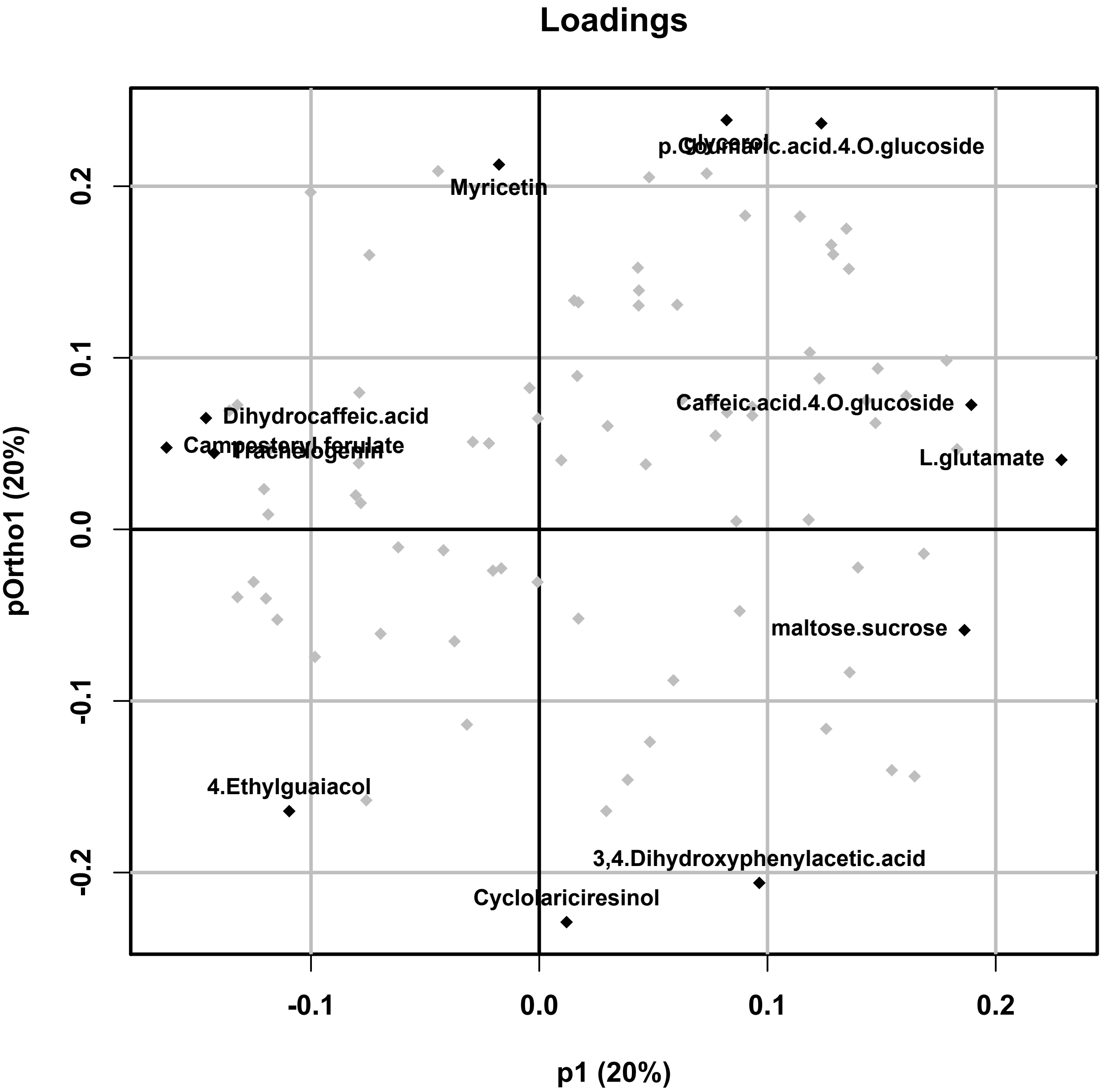

Supplementary Fig. S15. OPLS-DA model of root exudate samples of 140R rootstocks sampled at 6 h. Loading plot of OPLS-DA model (1P+1O, R^2X(cum)= 0.324, R^2Y(cum)= 0.998, Q^2(cum)= 0.734, RMSEE= 0.029; scaling: mean-centering and unit variance scaling) of root exudates collected at 6 h from Fe-deficient and Fe-sufficient 140R microcuttings.

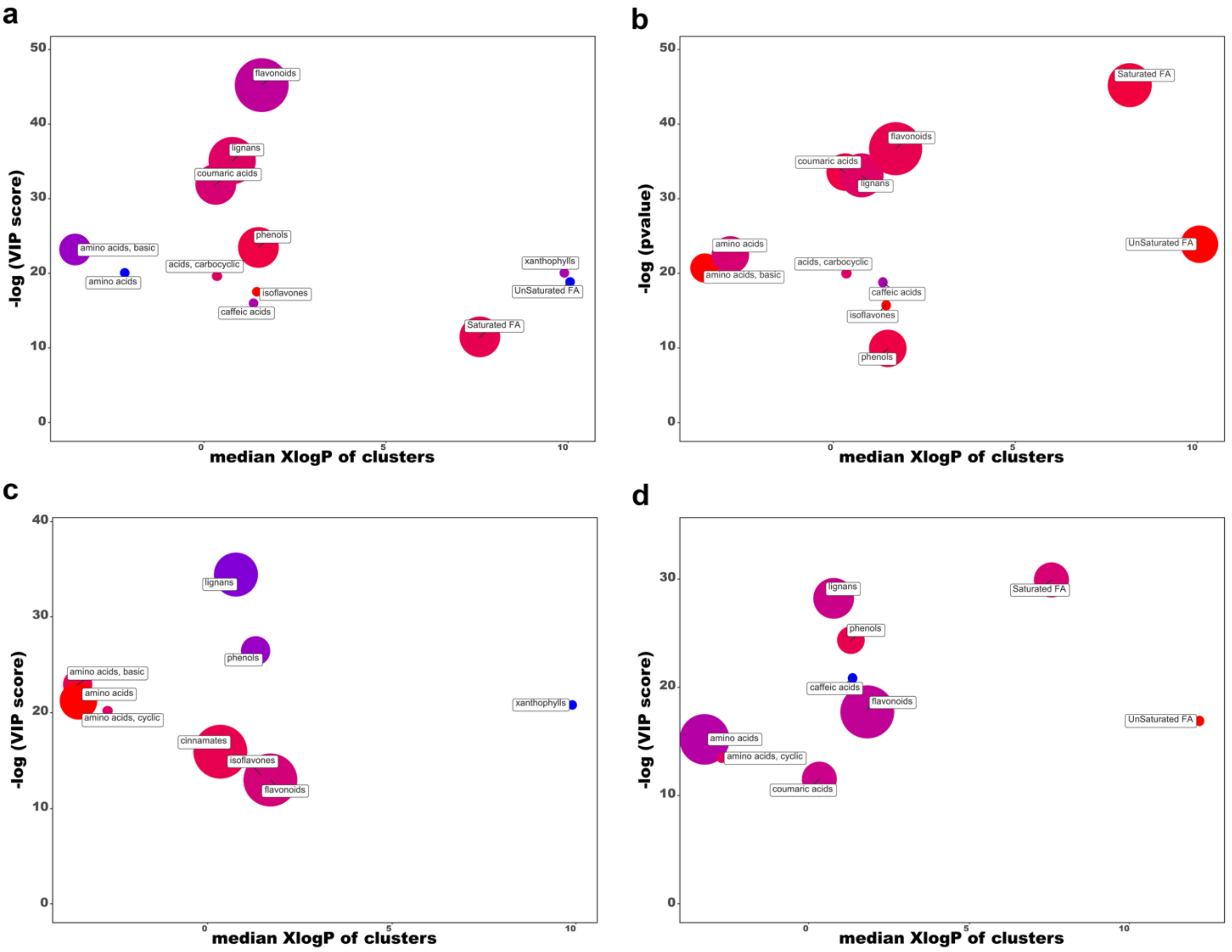

Supplementary Fig. S16. Plots from Chemical similarity enrichment analysis. Chemical similarity enrichment analysis based on VIP and fold-change (Fe+ *vs* Fe-) values of the metabolites identified **a** in Ramsey at 3 h, **b** in 140R at 3 h, **c** in Ramsey at 6 h and **d** in 140R at 6 h. Colour is according to proportion of increased or decreased compounds (red = increased, blue = decreased, pink = mixed) in each cluster. X-axis presents partition coefficient (XlogP values) of metabolite clusters, and the y-axis indicates the level of statistical significance by Kolmogorov–Smirnov test based on VIP scores.

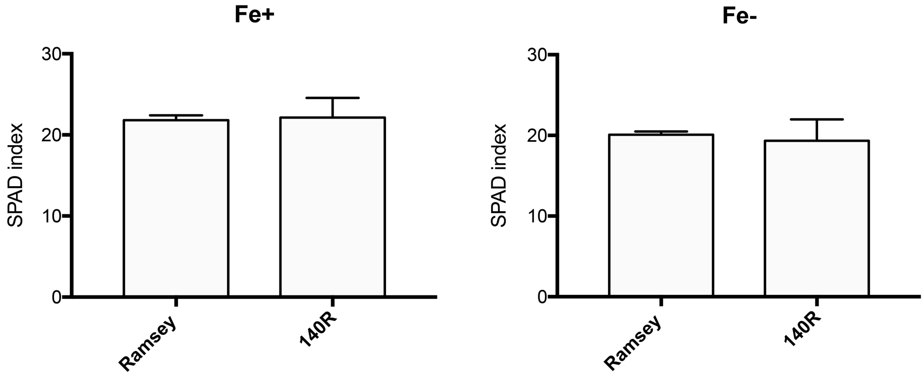

Supplementary Fig. S17. Comparison of SPAD index values between Ramsey and 140R microcuttings. SPAD values of Ramsey and 140R microcuttings grown in presence (Fe+) and in absence (Fe-) of Fe. The statistical significance was determined by Student's t-test (mean ± SE, n= 3 independent growth experiments).

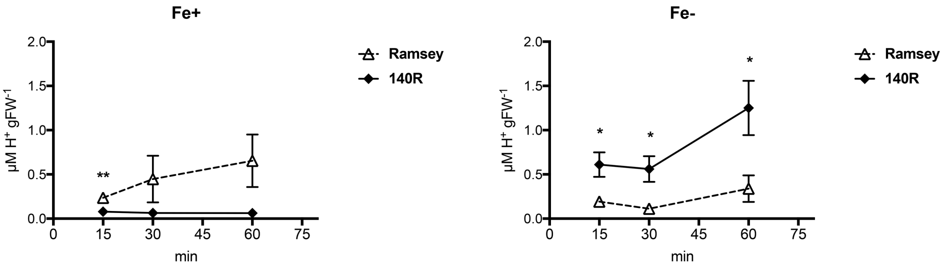

Supplementary Fig. S18. Time-course of root H^+ release. Comparison of H^+ extrusion by roots of Ramsey and 140R measured at 15, 30 and 60 min grown under Fe-sufficient and Fe-deficient condition. The statistical significance was determined by Student's t-test (mean ± SE, n= 4 plants, * $P < 0.05$. **, $P < 0.01$).
